# Topology-Driven Mechanical Tuning of FDM-Printed TPU Tubular Lattices with Auxetic Architectures

**DOI:** 10.3390/mi17091092

**Published:** 2026-09-17

**Authors:** Yun Zhai, Shenhui Sang, Yu Liu, David Hui, Depeng Shang

**Affiliations:** 1 School of Mechanical Engineering, Dalian Jiaotong University, Dalian 116028, China; yzhai@djtu.edu.cn (Y.Z.);; 2 Department of Mechanical Engineering, University of New Orleans, New Orleans, LA 70148, USA; dhui@lsuneworleans.edu; 3 Department of Orthopedics, Affiliated Zhongshan Hospital of Dalian University, Dalian 116001, China

**Keywords:** fused deposition modeling, thermoplastic polyurethane elastomer, negative Poisson’s ratio structures, tubular lattices

## Abstract

Auxetic tubular lattices provide a potential structural platform for simultaneously tuning radial support, axial dimensional stability, and bending flexibility; however, the coupled effects of topology and geometric parameters on these mechanical responses remain insufficiently understood. In this study, a topology-driven design framework was established for fused deposition modeling (FDM)-printed thermoplastic polyurethane (TPU) tubular lattices. Five auxetic topologies were designed by regulating curvature polarity and structural symmetry, while planar porosity and wall thickness were selected as geometric control parameters. Numerical simulations combined with experimental validation were employed to systematically evaluate axial bending stiffness, radial support force, Poisson’s ratio, and stress distribution. The results demonstrate that geometric curvature promotes radial load transfer through an arch-like mechanism and contributes to stress redistribution, whereas structural symmetry strongly influences axial compliance and auxetic deformation. Planar porosity acts as the dominant macroscopic tuning parameter for overall mechanical performance, while wall thickness provides a secondary fine-tuning mechanism with greater sensitivity to local stress variation. Based on these findings, a hierarchical design strategy integrating topology, planar porosity, and wall thickness is proposed, providing a generalizable approach for tailoring the mechanical performance of FDM-printed elastomeric tubular lattices.

## 1. Introduction

Cardiovascular disease (CVD) remains the leading cause of mortality and disability worldwide, with its global disease burden continuing to increase annually [1]. Atherosclerosis, the primary pathological basis of most CVDs, progressively causes vascular stenosis and occlusion through plaque accumulation [2,3]. Vascular stents have become one of the most widely used interventional treatments by providing radial support to restore vessel patency and improve blood flow [4]. An ideal vascular stent should simultaneously provide sufficient radial strength, excellent flexibility, and minimal axial recoil to ensure structural support, deliverability, implantation accuracy, and long-term stability [5,6,7,8]. However, these mechanical requirements are inherently coupled, and improving one property often compromises another. Therefore, achieving a balanced optimization of radial support, flexibility, and axial stability through structural design and material selection remains a major challenge in vascular stent research.

Currently, clinically applied vascular stents are primarily fabricated from metallic or polymeric materials [9]. Metallic stents, including iron based, magnesium, and cobalt chromium alloys, possess excellent mechanical strength but generally exhibit limited flexibility and poor conformability to tortuous vascular anatomies [10,11,12]. To improve flexibility while maintaining radial support, ultrathin strut designs have been widely adopted and have demonstrated superior deliverability in complex lesions such as tortuous and bifurcation vessels [13,14]. However, these designs often suffer from insufficient radial strength and increased recoil in chronic total occlusion and severely calcified lesions [15,16]. In addition, the permanent implantation of metallic stents may induce persistent inflammation and delayed endothelialization, thereby increasing the risk of late thrombosis [17,18]. Although biodegradable metallic stents partially alleviate these limitations, they remain challenged by degradation related issues such as metal ion release, gas generation, and premature structural failure [19]. Consequently, increasing attention has shifted toward polymer-based stents because of their superior biomechanical compatibility and compliance with native vessels [20,21]. The inherently low elastic modulus of polymeric materials provides compliance closer to that of native blood vessels. However, compared with metallic stents, polymer stents inherently exhibit insufficient radial support due to their low elastic modulus [22]. Increasing wall thickness, as adopted in the Absorb bioresorbable vascular scaffold, can partially compensate for this limitation, but inevitably compromises flexibility and increases thrombosis risk [23]. Therefore, improving the mechanical performance of polymer stents requires more than macroscopic dimensional adjustment. In this study, a topology-driven design strategy is proposed to enhance radial support by optimizing the unit cell architecture while maintaining structural flexibility.

The continuous evolution of structural design has been a major driving force in the advancement of vascular stents, with structural optimization progressively improving overall mechanical performance toward the ideal stent concept [24]. Conventional closed cell structures provide high radial support but limited flexibility, whereas open cell structures improve bending compliance at the expense of supporting strength [25]. To balance these conflicting requirements, researchers have proposed various hybrid topological designs. Representative studies have demonstrated that hybrid open closed cell configurations based on rhombic topologies can simultaneously improve radial support and flexibility without substantially increasing wall thickness [26,27,28]. Besides the overall lattice topology, the design of connecting bridges has also become an important strategy for regulating stent mechanics. Open cell stents commonly employ M-shaped, V-shaped, or U-shaped flexible connectors to improve bending compliance, whereas closed cell stents generally utilize short straight connectors to enhance structural rigidity [29,30,31]. Based on these design principles, different bridge configurations have been developed to achieve a better compromise between flexibility and radial support [32]. For polymer stents with relatively low elastic modulus, straight strut and closed loop designs have also been adopted to compensate for the limited intrinsic material strength [6,33]. Furthermore, different unit geometries exhibit distinct mechanical characteristics, highlighting the critical role of topology in regulating stent performance [34]. Overall, previous studies have primarily focused on optimizing macroscopic topology and connecting bridge configurations to improve the balance among radial support, flexibility, and axial stability. However, the mechanical roles of microscopic geometric features, including curvature polarity, curvature evolution, and the structural symmetry of supporting ring boundaries, remain insufficiently understood and lack systematic quantitative investigation.

Notably, female patients exhibit smaller and anatomically tortuous access vessels, imposing higher demands on the operational accuracy of stent implantation [35,36]. Conventional stent structures with a positive Poisson’s ratio, such as rhombic and hexagonal unit cells, generally exhibit axial recoil during balloon expansion, which may reduce implantation accuracy and increase the risks of vascular injury, thrombosis, and restenosis [31,37]. Structural optimization within positive Poisson’s ratio designs often involves a tradeoff among axial recoil, bending flexibility, and radial support, making it difficult to improve one property without compromising another [7,31]. In contrast, auxetic structures with a negative Poisson’s ratio have attracted increasing attention because they can simultaneously reduce axial recoil while maintaining favorable flexibility and radial support [38]. Representative studies have demonstrated the potential of negative Poisson’s ratio designs for vascular stents. Chen et al. optimized a concave hexagonal auxetic topology and achieved excellent axial and radial mechanical performance with good flexibility, even at low surface coverage ratios [39]. Teong et al. optimized a star shaped reentrant auxetic stent and reported improved deformation uniformity and vessel conformity through geometric parameter optimization [40]. Overall, these studies have demonstrated the considerable potential of auxetic topologies for vascular stents. However, few studies have systematically employed topological parameter design to quantitatively coordinate the three key mechanical properties of radial support, bending flexibility, and axial recoil within a unified design framework.

In this study, five polymer stent topologies were investigated, including flat edge and arc composite concave (FEA) stents, fully smooth bidirectional concave (FSB) stents, concave convex arc composite (CCA) stents, unidirectional arc concave (UAC) stents, and four angled star-shaped (FAS) stents. The stents were designed using computer aided design software and fabricated by fused deposition modeling. Planar porosity and wall thickness were selected as the key design variables, while numerical simulations and experimental validation were combined to evaluate the axial flexibility, radial support, and Poisson’s ratio of the five configurations. Based on these results, the effects of topological geometry and structural parameters on the mechanical behavior of the stent structures were systematically analyzed, providing a basis for the topology-driven mechanical design of FDM-printed elastomeric tubular lattices.

## 2. Materials and Methods

### 2.1. Biomimetic Topological Unit Cell Design and Array Modeling Logic

**Figure 1:**
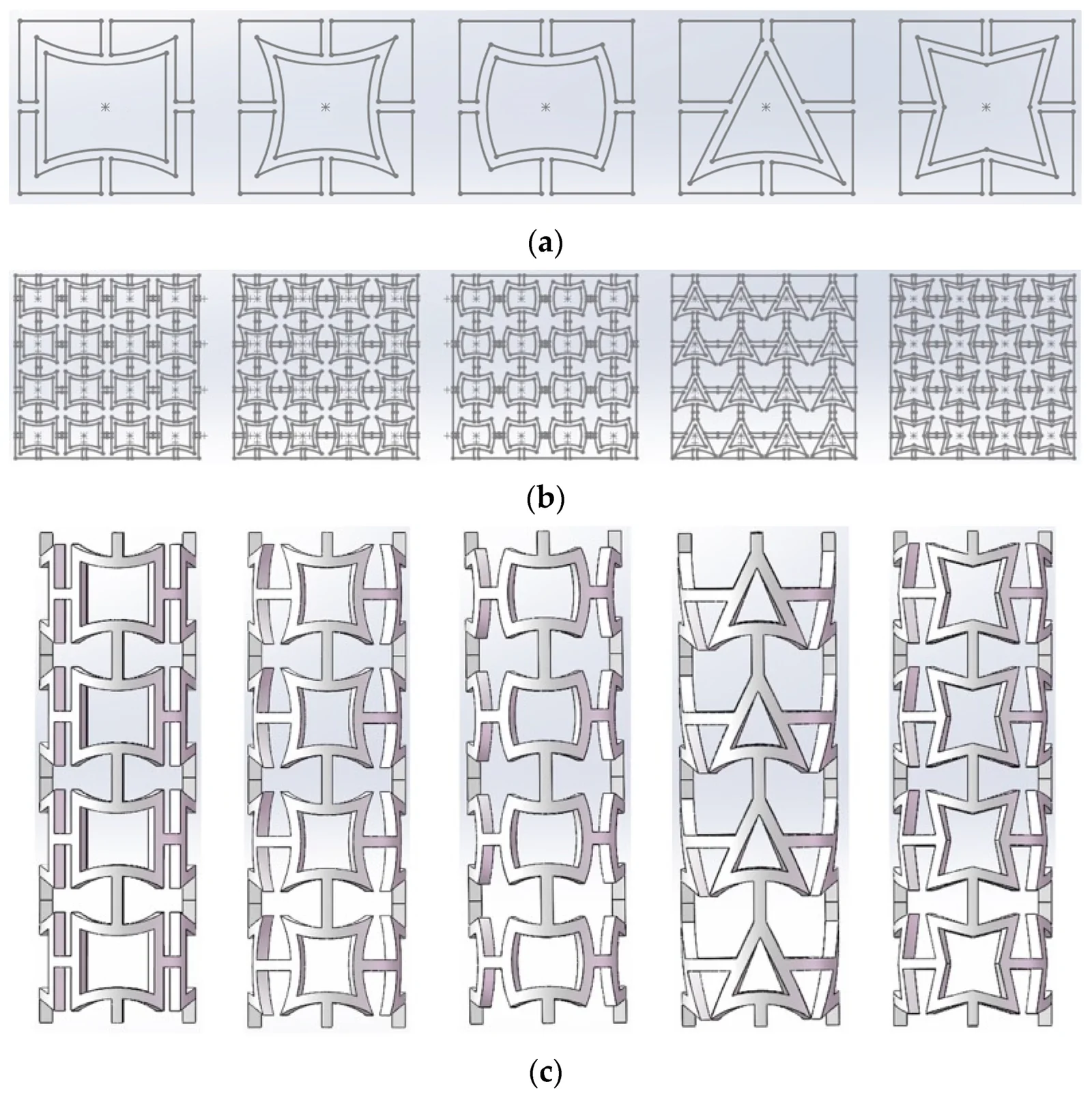
Design hierarchy and geometric modeling workflow of five topological stents: (**a**) Basic unit cell configurations. From left to right: FEA structure, FSB structure, CCA structure, UAC structure, and FAS structure; (**b**) 4 × 4 array-based planar lattice configurations. From left to right: array arrangement of the FEA structure, FSB structure, CCA structure, UAC structure, and FAS structure; (**c**) Three-dimensional tubular stent models formed by circumferential rolling around the central axis. From left to right: FEA stent, FSB stent, CCA stent, UAC stent, and FAS stent.

The mechanical behavior of vascular stents is primarily governed by the geometry of their unit cells. In this study, five representative topological configurations were designed, including FEA, FSB, CCA, UAC, and FAS stents. The FAS stent was selected as the reference configuration, from which four modified unit cells were derived by varying the curvature polarity and longitudinal symmetry of the supporting struts. Specifically, the FEA stent incorporates transverse straight edges and longitudinal concave arcs; the FSB stent adopts fully smooth bidirectional concave arcs; the CCA stent combines transverse convex arcs with longitudinal concave arcs; and the UAC stent retains a single longitudinal concave arc. The five unit-cell geometries are illustrated in Figure 1a.

The unit cells were periodically arranged into a 4 × 4 planar array with overall dimensions of 37.68 mm × 37.68 mm, as shown in Figure 1b. To investigate the effects of geometric parameters on stent mechanics, planar porosity (55%, 60%, 65%, and 70%) and wall thickness (1.0, 1.2, 1.4, and 1.6 mm) were selected as the two design variables. The specimen length was fixed at 37.68 mm, while the radius of all arc segments was maintained at 8 mm. The outer diameter of all stent structures was uniformly set to 12 mm with reference to the typical diameter range of large arteries, including the brachiocephalic trunk, suprarenal abdominal aorta, and other large peripheral arteries [41]. This geometric scale was primarily adopted as a proof-of-concept condition to enable systematic comparison of the mechanical responses and tunability of different auxetic topologies under a consistent dimensional framework, rather than to represent the final dimensions of a clinically deployable stent. Further translation toward clinical application will require geometry optimization according to specific vascular anatomy, together with the systematic evaluation of delivery compatibility, biocompatibility, and long-term mechanical reliability. Three-dimensional stent models were established in SolidWorks 2022 (Dassault Systèmes SolidWorks Corp., Waltham, MA, USA) by circumferentially rolling the planar lattices into tubular geometries with the prescribed wall thickness and planar porosity, as illustrated in Figure 1c. This modeling strategy ensured geometric continuity and provided a consistent basis for the subsequent finite element analysis.

### 2.2. Intrinsic Mechanical Characterization of Thermoplastic Polyurethane

Thermoplastic polyurethane (TPU) exhibits high elasticity, high tensile strength, good low-temperature adaptability, and excellent wear and corrosion resistance, and has been widely used in medical devices such as breast implants, catheters, and artificial heart valve leaflets [42]. In addition, TPU generally demonstrates good blood compatibility and relatively high stability in biological environments, while its long-term service behavior is mainly governed by the polymer chemical composition and surrounding environmental conditions. Previous studies have shown that under accelerated hydrolytic aging conditions of 0.1 mol/L NaOH at 37 °C, 3D-printed TPU exhibited only slight surface-related chain-segment changes after three months of aging, while the overall molecular weight remained essentially unchanged. The Young’s modulus, elongation at break, and ultimate tensile strength measured along the printing-layer direction also remained relatively stable, indicating favorable hydrolytic stability and mechanical property retention [43]. Furthermore, under in vitro degradation conditions in phosphate-buffered saline (PBS) at 37 °C, TPU structures exhibited substantially lower mass loss than PCL/PU and PCL/PU/gelatin composite systems, further demonstrating the relatively low short-term hydrolytic degradation rate of TPU [44]. When TPU was combined with gelatin to form a nanofibrous coating for stent grafts, patency was maintained for up to 28 days in a porcine iliac artery model, demonstrating its potential for vascular tissue engineering applications [45]. Meanwhile, the mechanical and processing properties of TPU can be tailored by adjusting the composition of polyols and diisocyanates, as well as by optimizing additive manufacturing process parameters, thereby enabling compatibility with different fabrication techniques. After process optimization, its ultimate tensile strength and elongation at break can reach or even exceed those of conventionally injection-molded products [46].

Based on these considerations, thermoplastic polyurethane (TPU) was selected as the matrix material for fabricating the elastic stent components. Specifically, the feedstock used in this study was PolyFlex™ TPU90 filament (1.75 mm in diameter), which was extruded and manufactured by Polymaker (Changshu, China). The base resin of this filament is the Addigy^®^ series neat TPU supplied by Covestro AG (Leverkusen, Germany). The purity of the original TPU polymer is higher than 99 wt %, incorporating only a minute quantity of UV stabilizer without the inclusion of any fillers or recycled materials. The fundamental mechanical and physical properties of the as-received TPU90 material are systematically summarized in Table 1. Additionally, to facilitate the successful additive manufacturing of the complex overhanging stent architectures, a sacrificial support material was employed. The support filament was Raise3D-certified PolyLite™ PLA, extruded and produced by Polymaker (Changshu, China). This printing filament was synthesized from Ingeo™ 4032D pure polylactic acid (PLA) resin supplied by NatureWorks LLC (Plymouth, MN, USA). The raw PLA polymer exhibits a high purity exceeding 99.7 wt %, with a residual lactide monomer content of less than 0.3 wt %. The finished support filament contained less than 1 wt % organic colorant and was completely free of fillers, recycled materials, or plasticizers.

### 2.3. Finite Element Numerical Simulation Settings

#### 2.3.1. Single Cantilever Beam Bending Model and Mechanical Boundary Conditions

Bending flexibility is an important mechanical characteristic of tubular stent structures because it reflects their ability to accommodate curvature without excessive structural resistance [51]. In this study, a cantilever beam bending model was employed to quantitatively evaluate the bending flexibility of the five stent structures [8].

**Figure 2:**
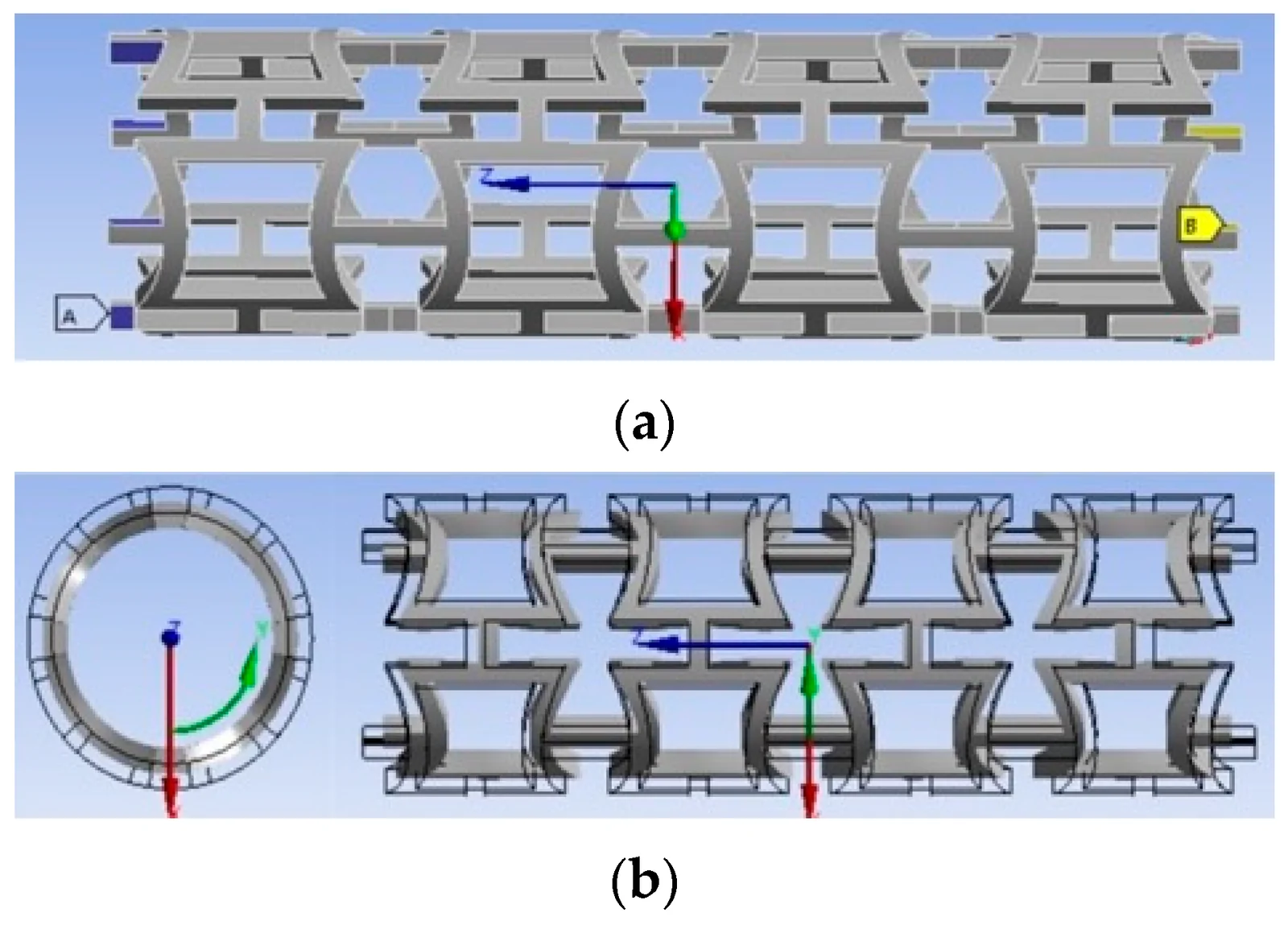
(**a**) Boundary condition setup for finite element simulation of unidirectional bending; (**b**) deformation before and after radial compression.

Since all five stent configurations had identical outer diameters, they were modeled as cylindrical structures with the same dimensions to ensure a consistent basis for comparing axial flexibility. The proximal end of the stent was fully constrained, while a vertical displacement of 5 mm was applied at the distal end to simulate cantilever bending. The equivalent bending stiffness $EI_{eq}$, which characterizes the resistance of the stent to bending deformation, was adopted as the primary evaluation index. During the simulations, the reaction force was extracted to calculate the equivalent bending stiffness, while the von Mises stress distribution was simultaneously analyzed to identify stress concentration regions and evaluate the structural safety of different topological configurations. To ensure computational accuracy, a global mesh with local refinement in critical load-bearing regions was employed. Multi-point constraints (MPCs) were applied to achieve coordinated structural deformation and accurate transfer of nodal degrees of freedom. In the static structural analysis, the left end of the stent was fully constrained, and a 5 mm displacement was applied at the reference point on the right end without imposing additional axial constraints. The loading and constraint conditions are illustrated in Figure 2a, where point A denotes the fixed end and point B represents the displacement loading point.

#### 2.3.2. Uniform Centripetal Radial Compression Model Under Cylindrical Coordinates

Radial support force is an important mechanical characteristic of stent structures and reflects their resistance to radial compression and structural collapse [52]. Insufficient radial support may limit structural stability, whereas excessive radial stiffness can compromise flexibility [53]. Therefore, coordinated regulation of radial support and bending compliance is an important consideration in the mechanical design of stent structures.

To evaluate the radial support performance of the stents, a controlled radial displacement loading method was employed. A uniform inward displacement was applied to reduce the stent radius from 6.0 mm to 5.4 mm, corresponding to a radial compression ratio of 10%. The total reaction force was extracted and normalized by the initial stent length to obtain the radial support force per unit length M, which was adopted as the primary evaluation index. In addition to the radial support force, the von Mises stress distribution was analyzed to identify stress concentration regions and assess the structural response under compression. The material properties and mesh settings were identical to those used in the bending simulations. A cylindrical coordinate system was established at the stent center to define the compression boundary conditions. The tangential and axial degrees of freedom of the internal nodes were constrained to eliminate non-radial deformation and rigid-body motion, while a radial displacement of 0.60 mm was applied uniformly to the outer surface. A global mesh with local refinement in critical regions was employed to improve the accuracy of stress and deformation predictions. The stent geometries before and after compression are shown in Figure 2b.

#### 2.3.3. Poisson’s Ratio-Based Characterization Method for Axial Response Under Radial Compression Conditions

Axial dimensional stability is an important characteristic of stent structures under radial deformation. Excessive axial contraction or elongation may alter the global geometry of the structure during radial compression or expansion [7,54]. To quantitatively characterize this response, Poisson’s ratio was introduced as a normalized metric for evaluating the coupling between radial deformation and axial dimensional change. After finishing the radial compression simulation, monitoring points were set at the edge nodes on both axial ends of the stent via the displacement probe function. The Poisson’s ratio γ was calculated from the axial deformation ΔL induced by radial deformation.

### 2.4. Experimental Protocol for Mechanical Characterization of Stents

**Figure 3:**
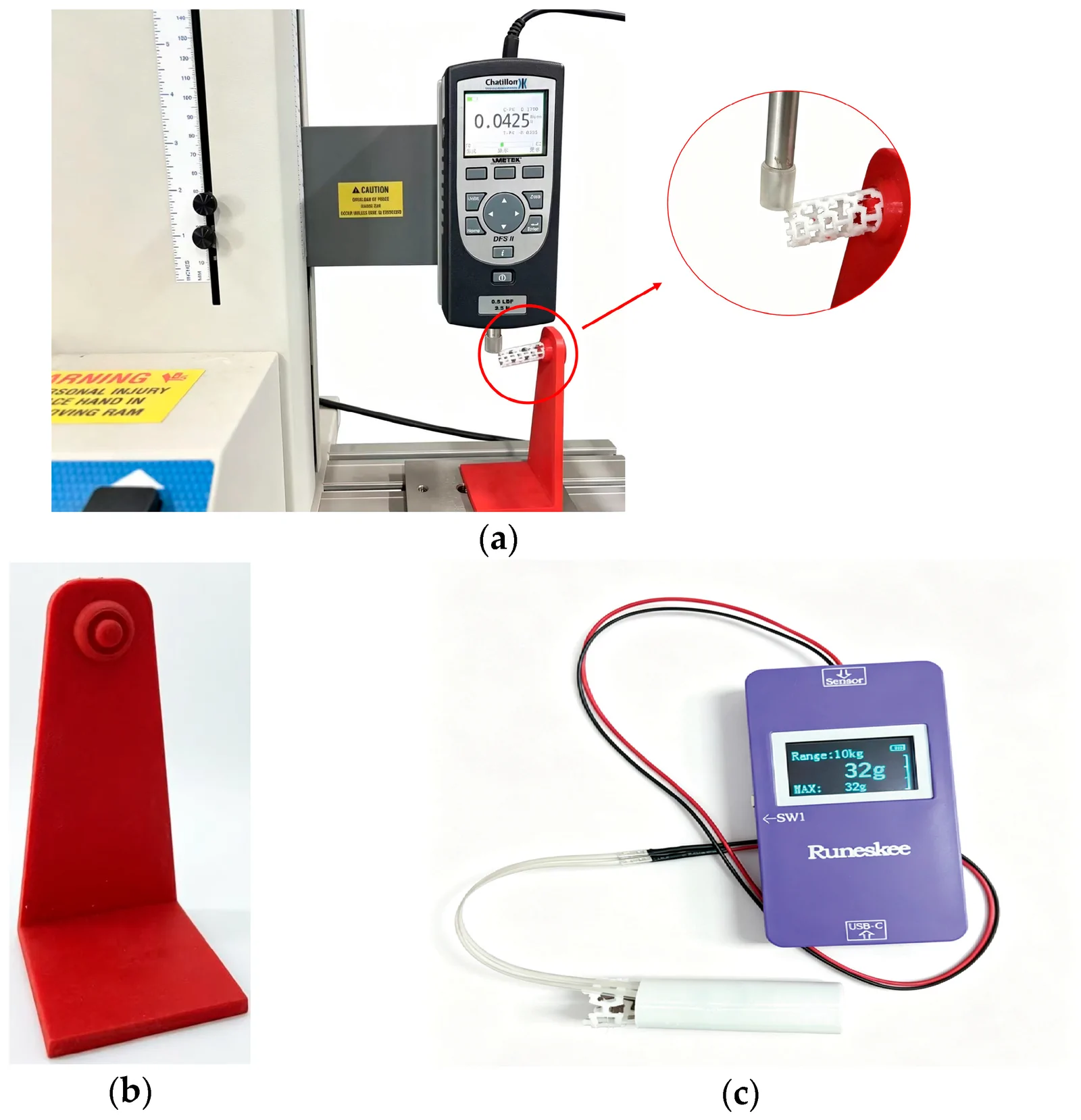
(**a**) Single-sided cantilever bending test; (**b**) stent fixation fixture; (**c**) radial compression test.

To evaluate the axial flexibility of the stents, standardized bending tests were conducted using a computer-controlled electronic universal testing machine composed of a single-column motorized force test stand (LTCM series, Chatillon, AMETEK, Greensburg, NY, USA) and a digital force gauge (DFS II, Chatillon, AMETEK, Greensburg, NY, USA) [8]. This universal testing platform, engineered for both tension and compression characterizations, was utilized to perform the single-sided cantilever bending evaluations. During the experiments, a loading indenter with a radius of 5 mm was employed, and the loading rate was set to 1 mm/min. A vertical displacement load of 5 mm was applied at a position 37.68 mm away from the fixed end of the stent. The fixture used for stent fixation is shown in Figure 3b, which provided rigid fixation at one end of the specimen. To ensure the repeatability and comparability of the experimental results, all specimens were tested under identical displacement-controlled loading conditions. The entire procedure of the single-sided cantilever bending test is illustrated in Figure 3a.

To evaluate the radial support performance of the stents under compression, a thin-film pressure sensing technique was introduced to establish an experimental characterization method for radial support force. During the test, the stent was first deployed into a simulated vessel and maintained in a compressed state. Owing to the elastic recovery of the stent material, the stent gradually expanded outward and continuously exerted support on the simulated vessel wall, thereby reproducing the mechanical interaction between the stent structure and the surrounding compliant tubular wall. The simulated vessel was fabricated from thermoplastic polyurethane with an elastic modulus of 3.4 MPa, an outer diameter of 12 mm, and an inner diameter of 10.8 mm. Previous studies have reported that the elastic modulus of this material is close to that of atherosclerotic coronary arteries (3.77±0.38 MPa), enabling it to reasonably reproduce the mechanical response of diseased vascular tissues [55]. A flexible thin-film pressure sensor (Model RX06A, RunesKee, Shenzhen Lojia Technology Co., Ltd., Shenzhen, China), coupled with a multichannel display and data acquisition system (Model CMCU-05A, RunesKee, Shenzhen Lojia Technology Co., Ltd., Shenzhen, China), was employed to continuously monitor and quantify the contact force exerted on the simulated vessel wall, thereby enabling quantitative assessment of the radial support performance of the five stent structures under constrained radial deformation. The experimental setup is shown in Figure 3c.

### 2.5. Fabrication of Stent Specimens Using Fused Deposition Modeling Technology

**Figure 4:**
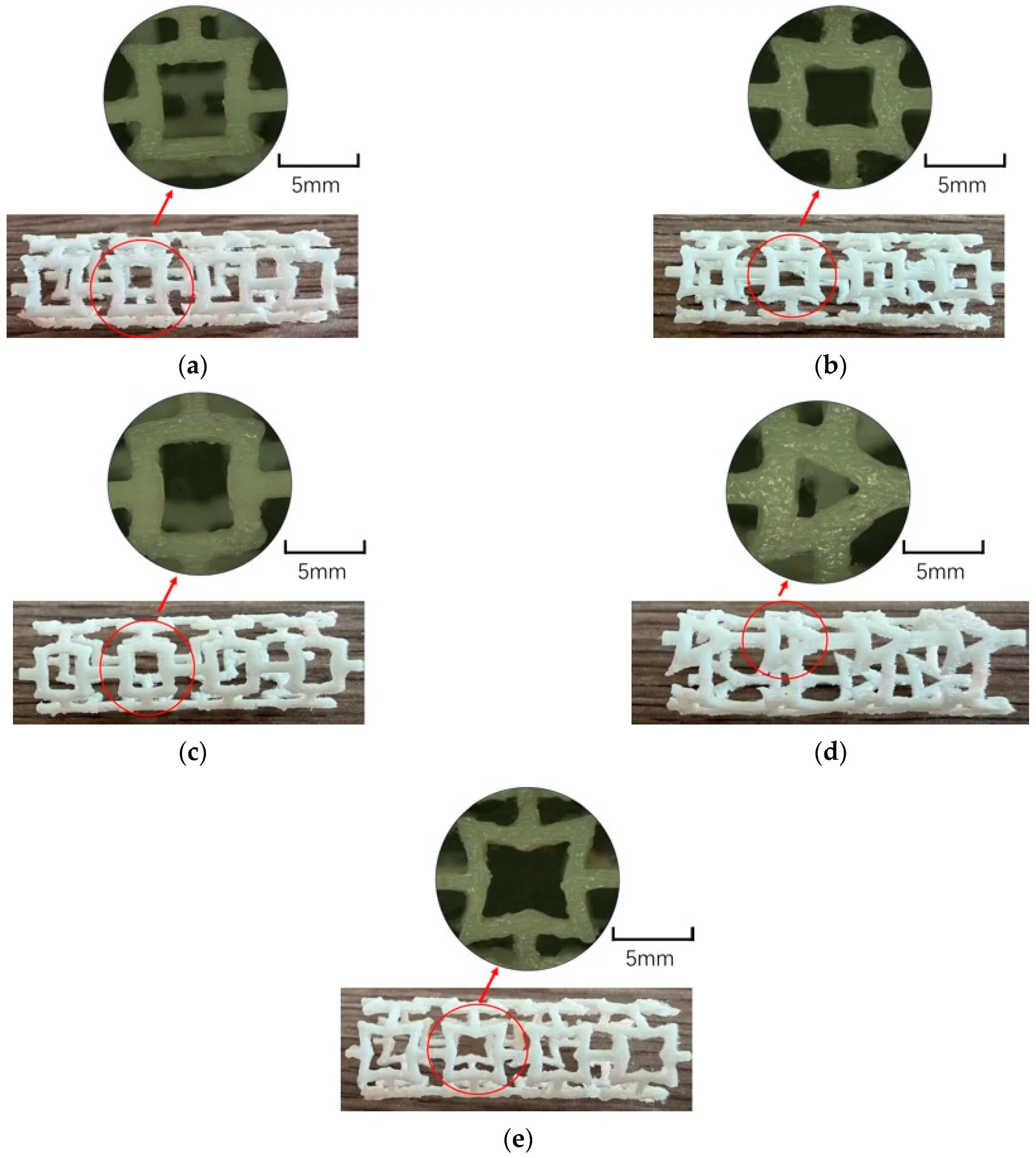
Multi-scale morphological characterization of TPU stents fabricated by fused deposition modeling (FDM): (**a**) FEA stent; (**b**) FSB stent; (**c**) CCA stent; (**d**) UAC stent; (**e**) FAS stent.

In this study, stent specimens were fabricated using fused deposition modeling (FDM) technology. A commercial E2 independent dual-extruder 3D printer, independently developed and manufactured by Shanghai Fusion Tech Co., Ltd. (Raise3D; global headquarters in Shanghai, China), was employed as the advanced additive manufacturing platform. The printing system provides a substantial build volume of 330 mm × 240 mm × 240 mm and maintains a high printing precision within the range of 0.05–0.10 mm. A dual-material printing strategy was adopted. The base material consisted of white thermoplastic polyurethane (TPU) filament with a diameter of 1.75 mm, extruded from the left nozzle at 230 °C. The support material was red polylactic acid (PLA), deposited from the right nozzle at 210 °C. The independent operation of the dual extruders effectively prevented cross-contamination between dissimilar materials, thereby ensuring improved surface quality of the fabricated stents. The printing parameters were set to a layer thickness of 0.15 mm and a fill density of 100%. Figure 4 presents macroscopic views of the five topological stent designs together with 3× magnified images of selected regions. The red circles indicate the regions selected for magnification, and the red arrows link each circled region to its corresponding enlarged view. The micrographs clearly reveal the layer-by-layer morphology inherent to the FDM process, the boundary formation quality, and the surface condition after removal of the support material. These observations provide a direct morphological basis for the subsequent analysis of performance deviations. The complete macroscopic views of the remaining stents are provided in Supplementary Material Figures S1 and S2.

## 3. Results and Discussion

### 3.1. Topology-Dependent Regulation of Axial Bending Flexibility

#### 3.1.1. Equivalent Stress Distribution Under Single-Side Bending

**Figure 5:**
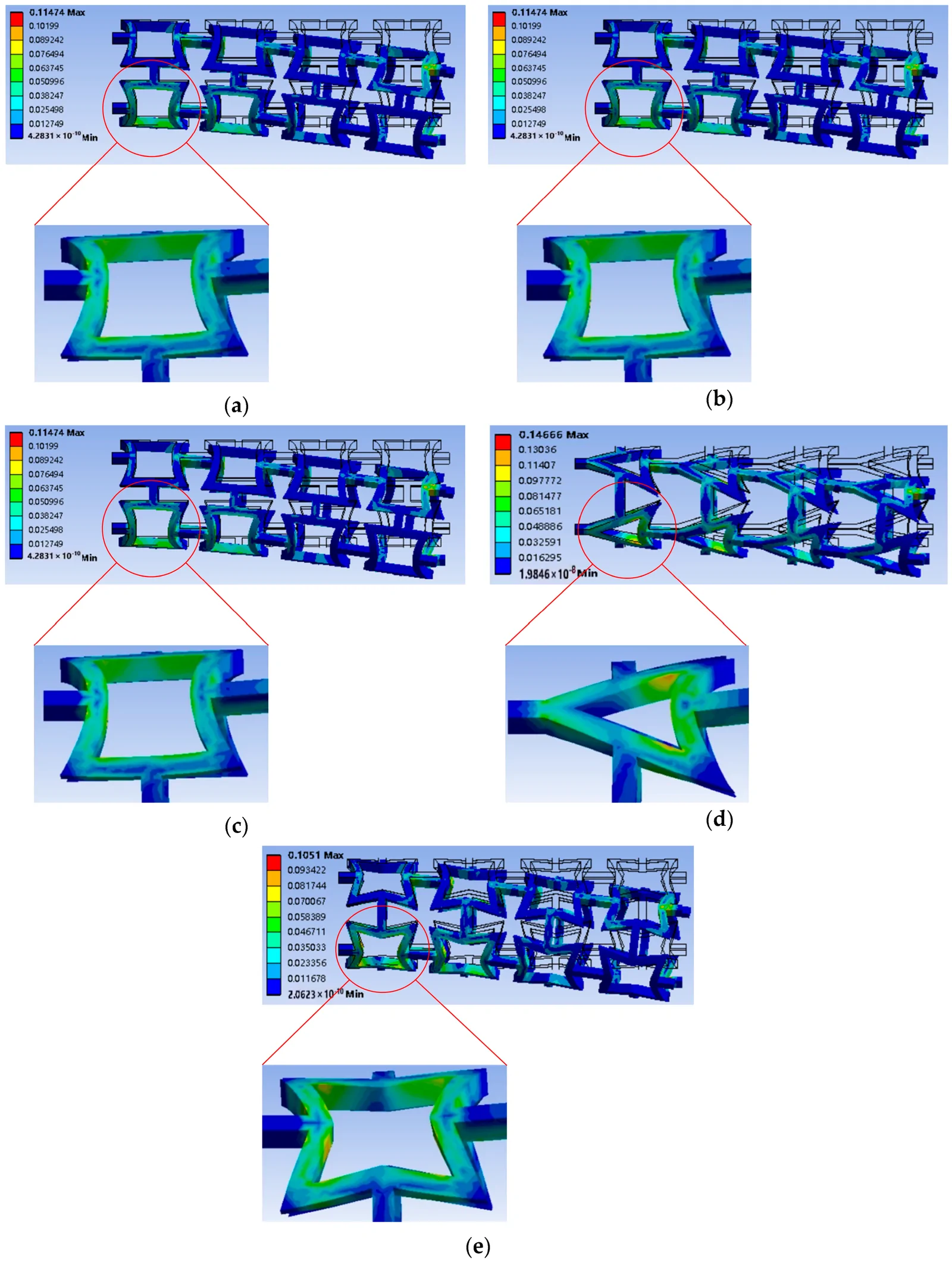
(**a**) Equivalent stress distribution of the FEA stent under bending; (**b**) equivalent stress distribution of the FSB stent under bending; (**c**) equivalent stress distribution of the CCA stent under bending; (**d**) equivalent stress distribution of the UAC stent under bending; (**e**) equivalent stress distribution of the FAS stent under bending.

This study quantitatively evaluated the von Mises stress distributions of five stent topologies under bending. Because each topology exhibited similar stress distribution patterns, a representative model with a planar porosity of 70% and a wall thickness of 1 mm was selected for analysis, as shown in Figure 5. The red circle marks the region of the magnified unit cell, and the black wireframe represents the undeformed configuration of the stent. The remaining results are presented in Supplementary Material Figures S3–S12. Under unilateral cantilever bending, the fully constrained proximal end and the 5 mm displacement applied at the distal end generated a bending moment gradient, producing tensile stress on the convex side and compressive stress on the concave side. Figure 5a–e shows that the peak von Mises stress was consistently concentrated near the fixed end and gradually decreased toward the free end, consistent with Euler–Bernoulli beam theory [56]. Accordingly, the first unit cell adjacent to the fixed boundary was selected for detailed stress analysis.

The enlarged views in Figure 5 further illustrate the influence of topology on stress distribution. The FEA stent maintained relatively good axial stability owing to its straight struts, although stress was mainly concentrated in the curved regions that accommodate bending deformation. In contrast, the FSB and CCA stents exhibited more uniform stress distributions. Their continuous curved geometries promote smoother load transfer, thereby reducing local stress concentration. The UAC stent, due to its geometric asymmetry and abrupt structural transitions, exhibited a pronounced “rigid rib” effect along the axial direction. Under imposed displacement, the lack of sufficient geometric rotational freedom for stress release forces the structure to accommodate deformation primarily through material-level tensile–compressive interactions, leading to elevated stress concentrations. This is reflected in the continuous high-stress regions observed along the straight struts. Finally, the FAS stent, as a typical negative Poisson’s ratio unit, demonstrated a highly localized stress distribution pattern. The applied load was strongly anchored at the re-entrant corners of the star-shaped geometry, resulting in distinct point-wise stress concentrations.

**Figure 6:**
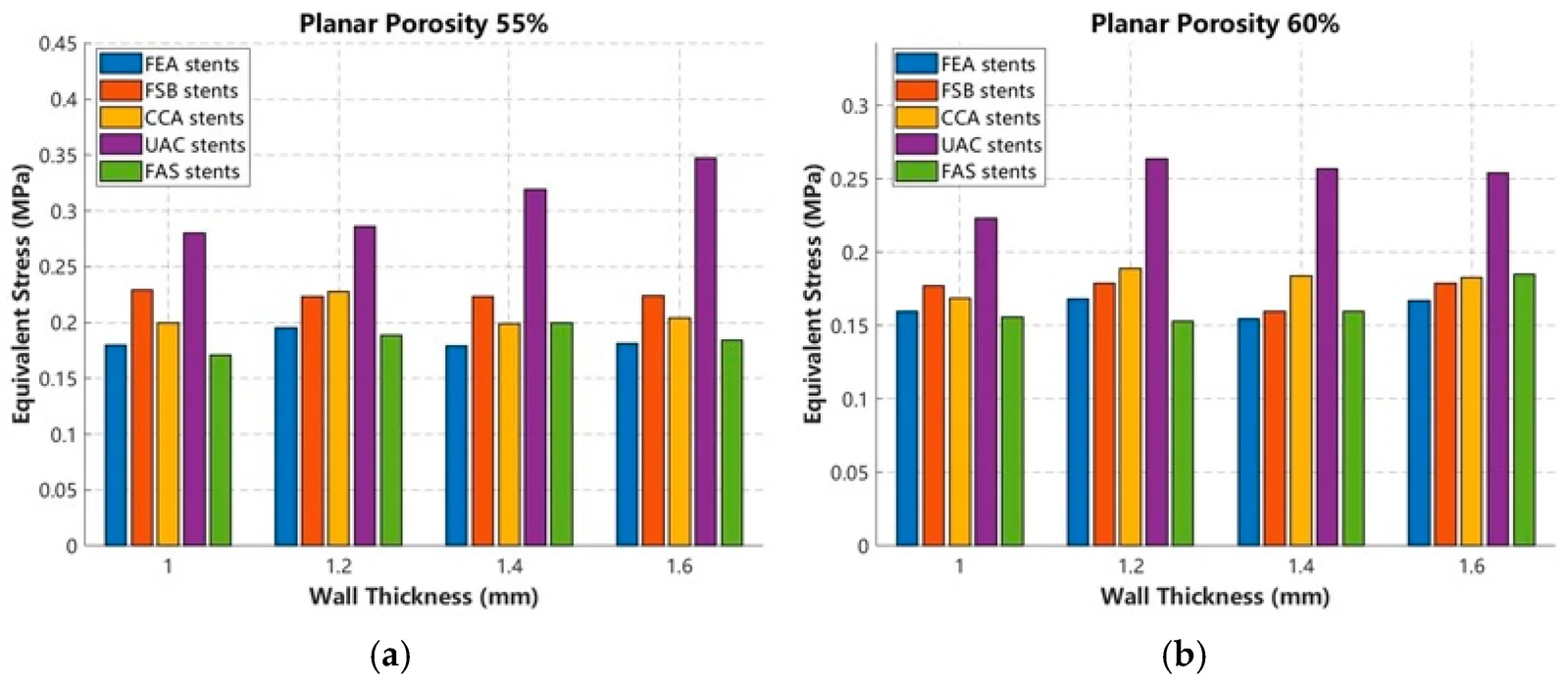

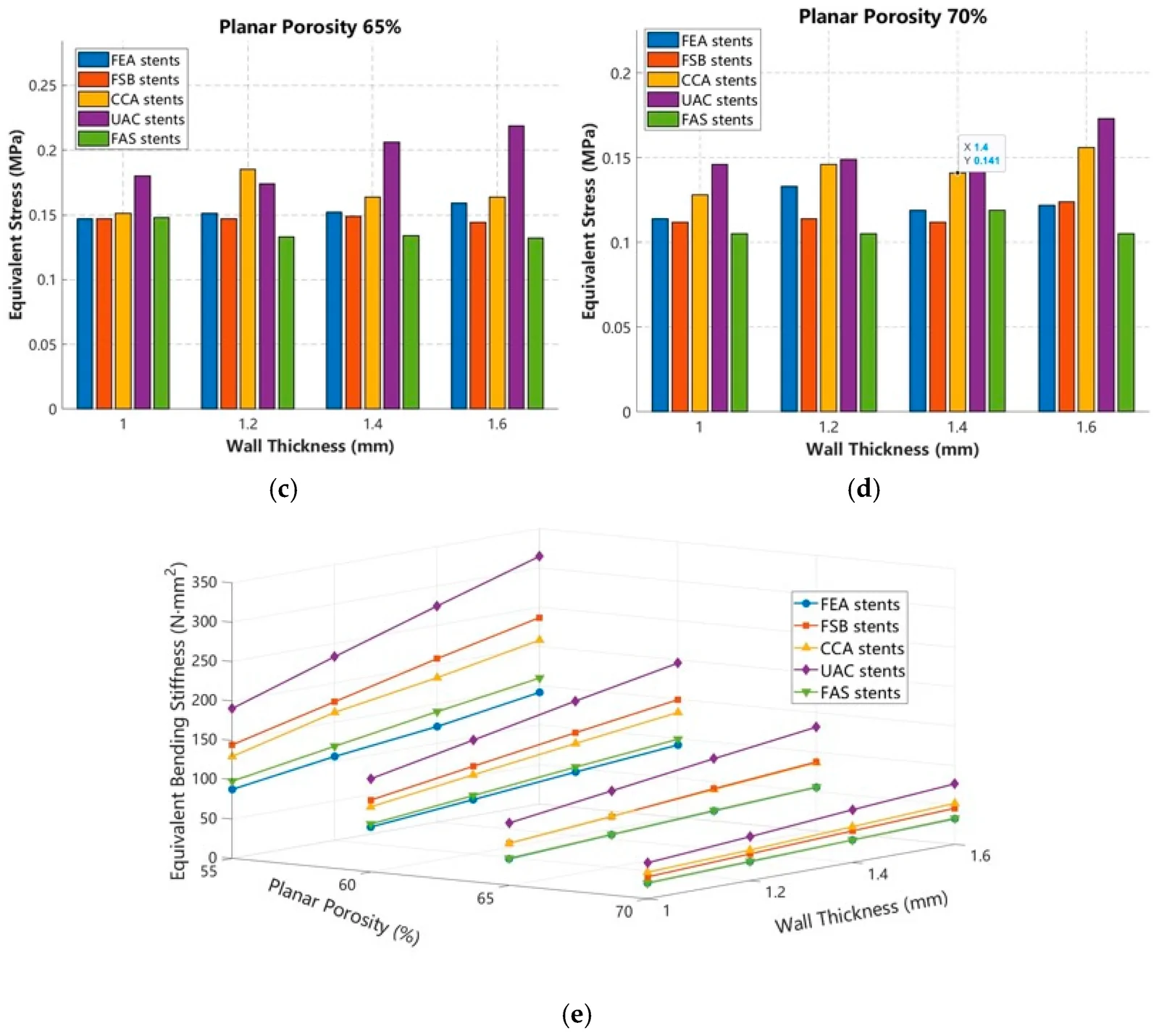
(**a**) Bar chart of maximum equivalent stress of stents with 55% planar porosity under bending; (**b**) bar chart of maximum equivalent stress of stents with 60% planar porosity under bending; (**c**) bar chart of maximum equivalent stress of stents with 65% planar porosity under bending; (**d**) bar chart of maximum equivalent stress of stents with 70% planar porosity under bending; (**e**) variation trend of equivalent bending stiffness of the five stent designs under different design parameters in the simulations.

To investigate the stress response characteristics of the topological stents under bending conditions and elucidate their relationship with geometric parameters, the maximum equivalent stress values corresponding to different combinations of planar porosity and wall thickness were extracted and summarized in the form of bar charts, as presented in Figure 6a–d. The overall trend indicates that the peak equivalent stress generally increases with increasing wall thickness. From a mechanical perspective, increasing wall thickness enhances the local section modulus of the supporting struts, thereby improving the overall deformation resistance of the structure under displacement controlled loading conditions. In contrast, increasing planar porosity reduces the volume fraction of solid material and provides greater deformation freedom for the unit cells, resulting in a gradual reduction in stress levels. Based on these stress evolution characteristics, it can be inferred that the proposed topological configurations are capable of regulating both the strain energy dissipation pathways and the structural safety margin against failure.

#### 3.1.2. Evolution of Equivalent Bending Stiffness with Planar Porosity and Wall Thickness

Using the probe function in ANSYS Workbench 2022 R1 (ANSYS, Inc., Canonsburg, PA, USA), the reaction force at the fixed end of each stent was extracted and used to calculate the equivalent bending stiffness of each structural configuration. The variation trends of bending stiffness for the five stent configurations under different design parameters are presented in Figure 6e. The results indicate that the axial equivalent bending stiffness generally increases with increasing wall thickness and decreases with increasing planar porosity. Moreover, the relationship between stiffness and wall thickness is approximately linear, which is consistent with the fundamental mechanical behavior of thin walled cylindrical structures. The stiffening effect induced by wall thickness is influenced by planar porosity. As porosity increases, the effective solid cross sectional area decreases, weakening the enhancement of the moment of inertia and consequently reducing the slope of the stiffness growth curves. When the planar porosity increased from 55% to 60%, a pronounced stepwise reduction in equivalent bending stiffness was observed for all stent configurations, with the UAC stent exhibiting the most significant decline. This phenomenon suggests a transition in the dominant deformation mechanism from shell dominated behavior to truss dominated behavior. At a relatively low porosity of 55%, the stent behaves similarly to a perforated thin walled tube, where the load is primarily sustained through axial tension and compression of the structural members, resulting in relatively high overall stiffness. When the porosity exceeds 60%, the slender struts increasingly deform through bending and rotational mechanisms at the joints, leading to a gradual reduction in the load bearing contribution of the shell effect and a corresponding decrease in structural stiffness.

From the perspective of structural topology, the flexibility of the five stent configurations followed the order: FEA stents > FAS stents > CCA stents > FSB stents > UAC stents. Analysis of the simulation results indicated that the geometric curvature of the transverse struts was one of the primary factors governing structural stiffness. Under bending loads, curved members generated an arch effect, whereby part of the external load was transformed into axial forces within the struts. Consequently, deformation was partially resisted through the axial stiffness of the material, leading to enhanced structural rigidity. Owing to this mechanism, the FSB stents and CCA stents exhibited higher equivalent bending stiffness than the FEA stents.

Furthermore, the simulation results demonstrated that inwardly concave transverse struts provided a more pronounced stiffening effect than outwardly convex geometries. During axial bending, the concave cells located on the compressive side tended to contract further, resulting in a more compact cellular configuration and an increase in structural resistance to deformation. In contrast, the outwardly convex transverse struts of the CCA stents gradually flattened under loading, causing their geometry to approach that of straight members and consequently reducing their bending resistance relative to the FSB stents.

A comparison between the FEA stents and UAC stents further revealed the influence of longitudinal concave symmetry on structural flexibility. Overall, the single-sided concave configuration exhibited substantially higher stiffness than the bidirectional concave configuration. In the FEA stents, the inwardly curved longitudinal struts on both sides can be mechanically regarded as pre-curved beams. When subjected to bending loads, these curved members generate additional bending moments due to geometric eccentricity, thereby facilitating deformation under relatively low external loads. In contrast, the UAC stents replace one concave side with a straight longitudinal strut, introducing a rigid constraint region. Because the line of action of the axial force nearly coincides with the centroidal axis of the straight member, the eccentricity is minimized, and deformation is primarily governed by axial tension and compression of the material, resulting in greater overall stiffness.

Among the bidirectional concave configurations, the FSB stents exhibited higher stiffness than the FAS stents. This difference can be attributed to the presence of sharp re-entrant corners in the FAS stents, which promoted stress concentration and localized rotational deformation under loading. In contrast, the continuous curved geometry of the FSB stents reduced local stress concentration and enabled more uniform load transfer throughout the structure, thereby enhancing the overall bending resistance.

### 3.2. Topology-Driven Mechanisms of Radial Support Performance

#### 3.2.1. Equivalent Stress Distribution Under Radial Compression

**Figure 7:**
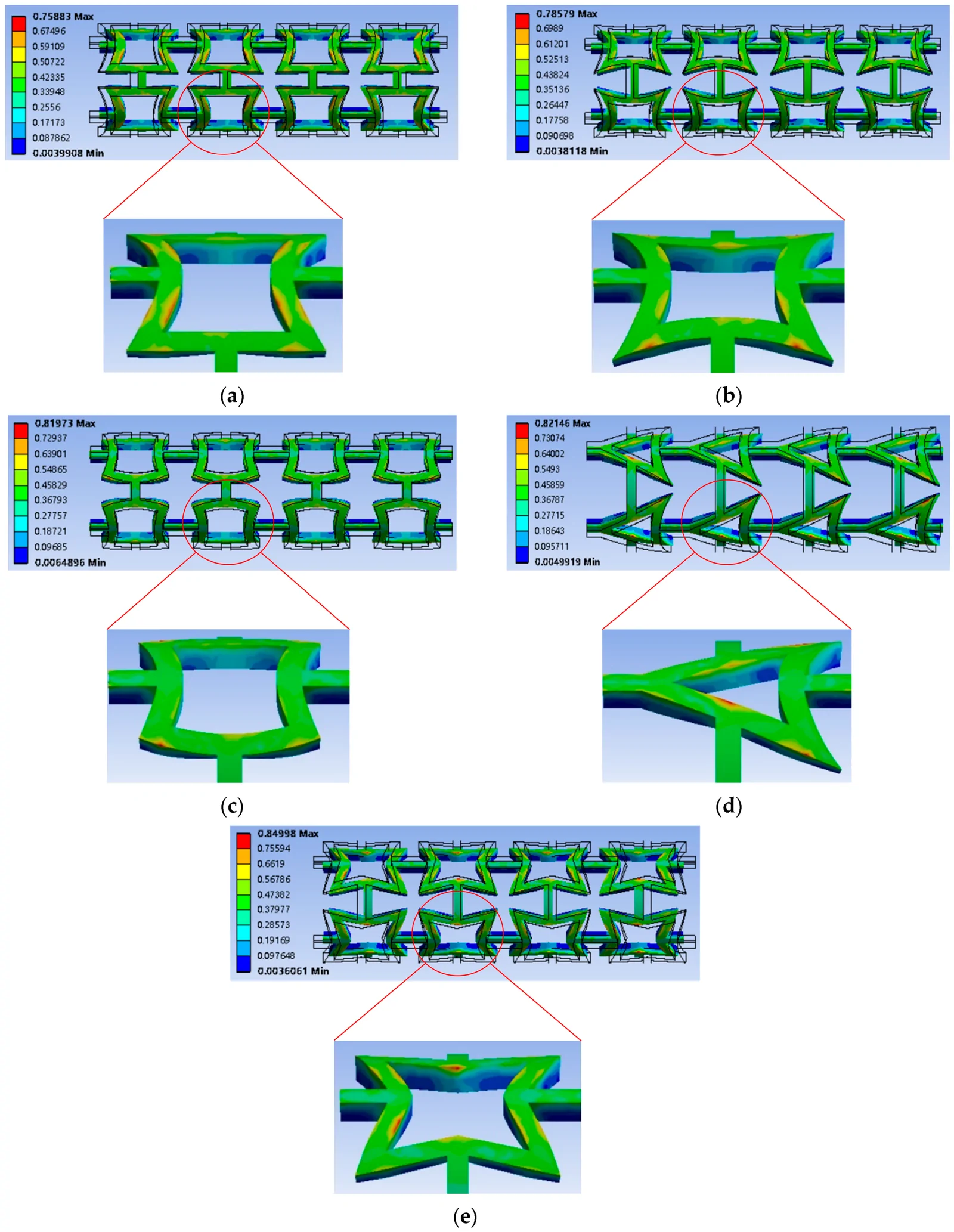
(**a**) Equivalent stress distribution of the FEA stent under compression; (**b**) equivalent stress distribution of the FSB stent under compression; (**c**) equivalent stress distribution of the CCA stent under compression; (**d**) equivalent stress distribution of the UAC stent under compression; (**e**) equivalent stress distribution of the FAS stent under compression.

This study quantitatively evaluates the evolution of von Mises stress in five topological stent configurations under compression conditions. Considering the high degree of self-similarity in stress distribution patterns within each topology, a representative specimen with a planar porosity of 70% and a wall thickness of 1 mm was selected for contour analysis, as shown in in Figure 7. The red circle marks the magnified unit cell, while the black wireframe represents the undeformed stent configuration. The remaining configurations and numerical results are provided in Supplementary Material Figures S3–S12.

Based on the stress contour analysis of unit cells located at the peak stress regions, different topological configurations appear to exhibit distinct load transfer characteristics. As illustrated in the enlarged views in Figure 7, the stress field of the FEA stent is mainly concentrated within the vertically oriented straight struts and the transition regions connecting the straight and curved segments. This distribution suggests that the straight edges may contribute to the formation of a relatively stable load-bearing framework, while the arc segments potentially facilitate stress redistribution and reduce abrupt stress gradients. The FSB stent exhibits a comparatively uniform stress distribution. The continuous curvature throughout the structure may promote a more even transfer of radial compressive loads, leading to reduced local stress accumulation. As a result, stress concentration regions appear less pronounced than in several other topologies. For the CCA stent, the alternating concave and convex curvatures appear to generate a mechanically complementary deformation pattern under radial compression. The stress contours show an alternating distribution along the geometric centerline, which is likely associated with the periodic curvature transitions within the unit cell. In contrast, the UAC stent exhibits relatively higher stress levels at specific geometric locations. This behavior may be related to the presence of geometric discontinuities and angular features, which can restrict local deformation and promote stress accumulation at the vertex regions during compression.

**Figure 8:**
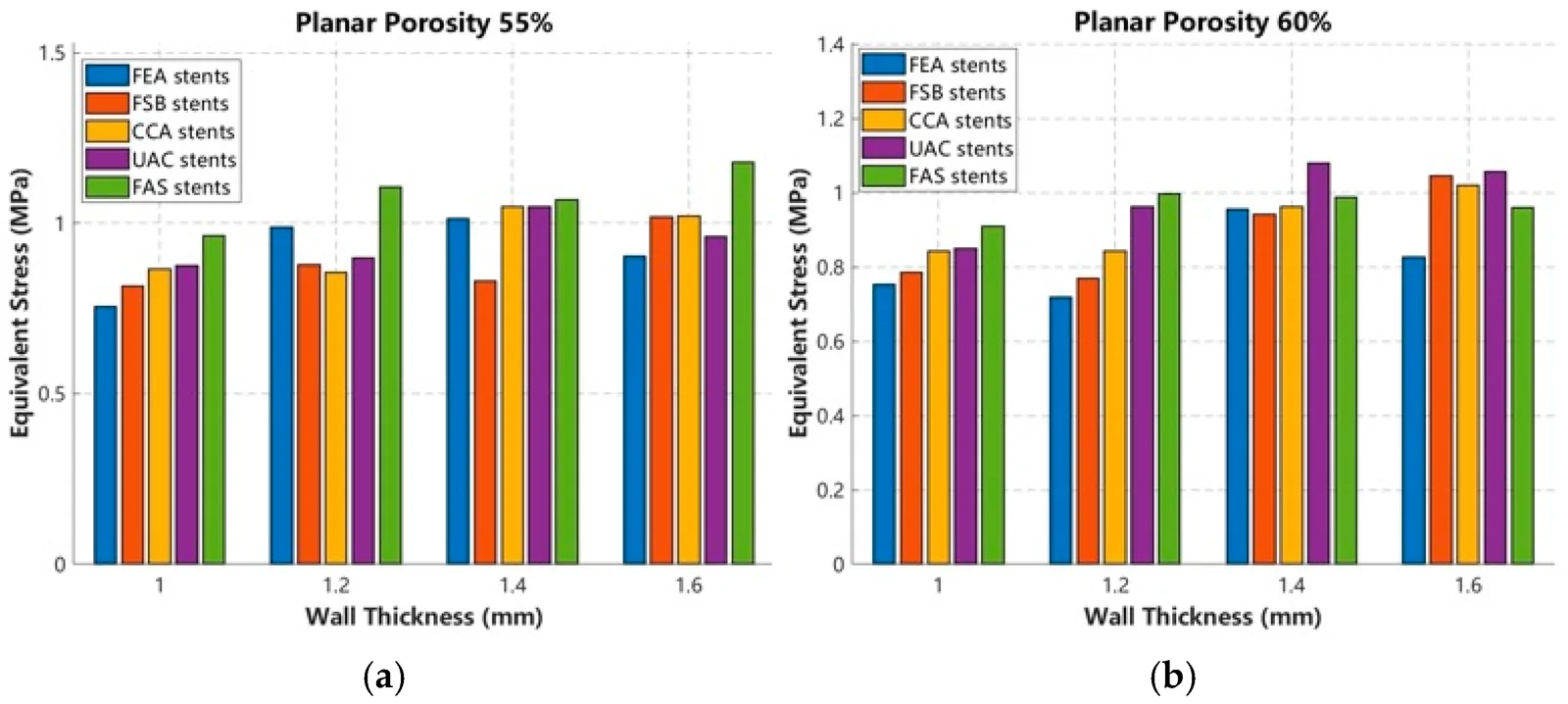

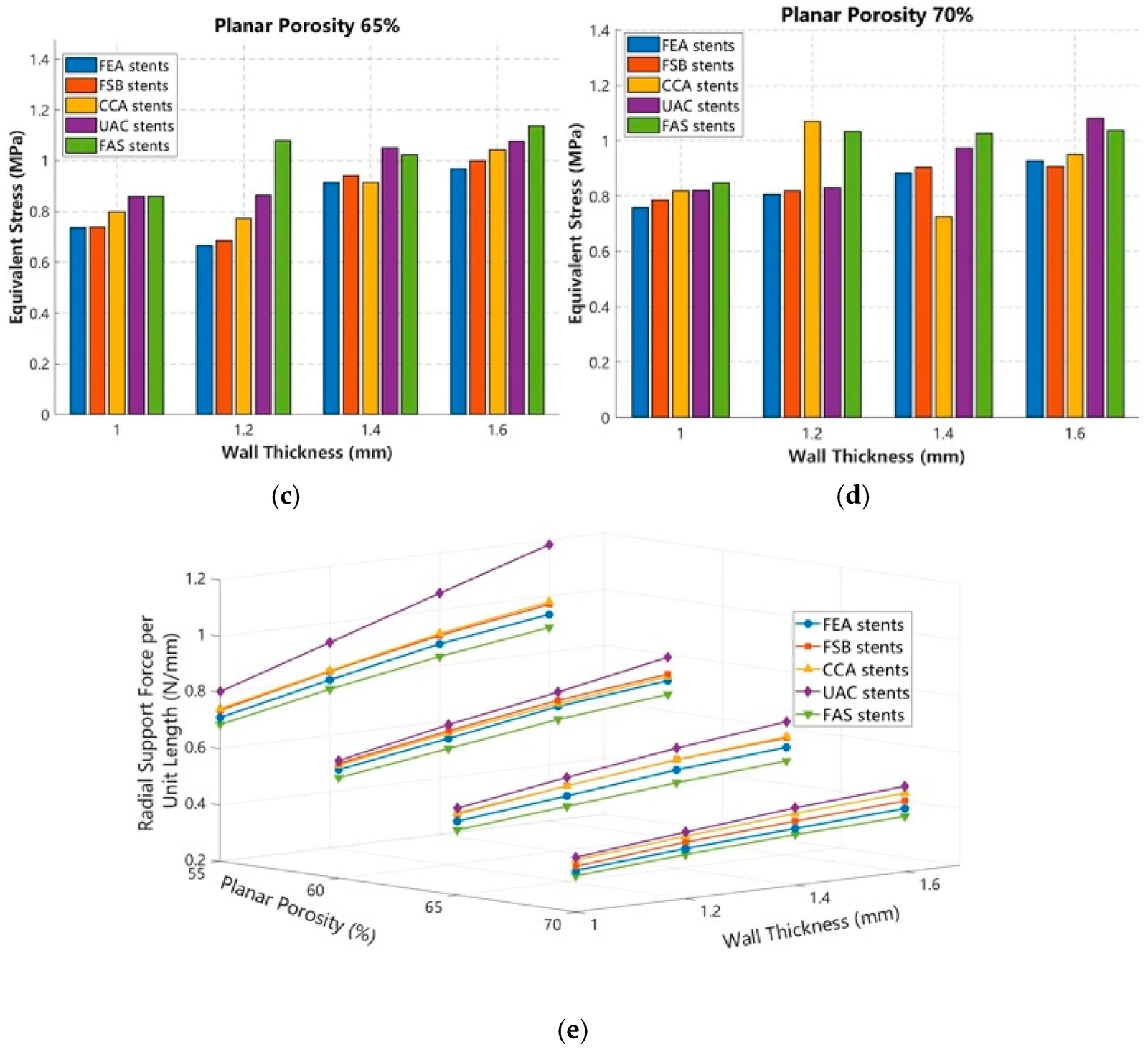
(**a**–**d**) Bar charts of the maximum equivalent stress under compression for stents with planar porosities of 55%, 60%, 65%, and 70%, respectively. (**e**) Variation trend of radial supporting force per unit length of the five stent designs under different design parameters in the simulations.

The FAS stent shows a distinct stress concentration pattern, with elevated stresses primarily located at the re-entrant corners of the star-shaped unit cell. Such a distribution suggests that localized rotational deformation may play an important role in the overall deformation response, whereas the remaining strut segments appear to experience comparatively lower strain energy levels. To further investigate the relationship between stress response and geometric parameters under radial compression, the maximum von Mises stress values were extracted for different combinations of planar porosity and wall thickness and are summarized in Figure 8a–d. Overall, the peak stress tends to increase with increasing wall thickness. This trend may be attributed to the increase in local structural stiffness and material volume fraction, which enhances resistance to radial deformation and consequently elevates local stress levels. In comparison, variations in planar porosity appear to have a relatively limited influence on the peak equivalent stress. This observation suggests that under the present loading conditions, local geometric constraints within the unit cells may play a more important role in determining stress concentration than the overall openness of the lattice structure. Nevertheless, further experimental validation and more detailed mechanical analyses would be required to fully clarify the underlying load transfer mechanisms.

#### 3.2.2. Evolution of Radial Support Force per Unit Length with Porosity and Wall Thickness

The built-in probe tool within the ANSYS Static Structural module was utilized to collect reaction force data on the outer surface of five stent designs for multiple planar porosity-wall thickness matching schemes. After normalizing the reaction forces by the actual length of each stent, the radial support force per unit length was acquired. The processed data were subsequently used to generate the performance trends shown in Figure 8e.

Overall, the simulation results suggest that the radial support force per unit length of all five stent configurations tends to decrease with increasing planar porosity while exhibiting an approximately linear increase with wall thickness. In addition, the effect of wall thickness appears to become more pronounced at higher porosity levels. This trend may be associated with the reduction in effective material fraction caused by increased porosity, making wall-thickness-related reinforcement relatively more important for resisting radial deformation.

From a comparative perspective, the simulated radial support performance generally follows the order: UAC stents > CCA stents > FSB stents > FEA stents > FAS stents. Comparison among the FEA, FSB, and CCA stents suggests that the curvature distribution of the transverse struts may play an important role in determining radial support behavior. Structures incorporating curved members tend to exhibit higher radial support forces than those dominated by straight segments. In particular, convex curvature appears to provide a greater enhancement than purely concave geometries. This tendency may be related to an arch-like load transfer mechanism, in which a portion of the radial load is redistributed into axial compressive stresses along the struts, potentially improving load-bearing efficiency. Although the FSB stents also incorporate continuous curvature, their inwardly concave geometry may facilitate localized inward deformation under compression, which could partially offset the beneficial effect of curvature on radial stiffness. As a result, their radial support performance appears slightly lower than that of the CCA stents.

A comparison between the FEA and UAC stents further suggests that the number and symmetry of longitudinal concave features may influence the compression response. The single-sided concave configuration generally exhibits higher radial support than the double-sided concave configuration. This observation may be associated with the preservation of a more continuous circumferential load-transfer path in the UAC stents, which could help limit coordinated inward deformation of neighboring unit cells during compression. Furthermore, within the group of bidirectional concave topologies, the FSB stents exhibit higher radial stiffness than the FAS stents. One possible explanation is that the sharp re-entrant corners in the FAS topology introduce localized stress concentration and deformation localization under loading. In contrast, the smooth curved geometry of the FSB stents appears to promote a more uniform stress distribution and load transfer process, which may contribute to the higher radial reaction force observed under equivalent deformation conditions.

### 3.3. Axial Dimensional Stability Driven by the Negative Poisson’s Ratio Effect

**Figure 9:**
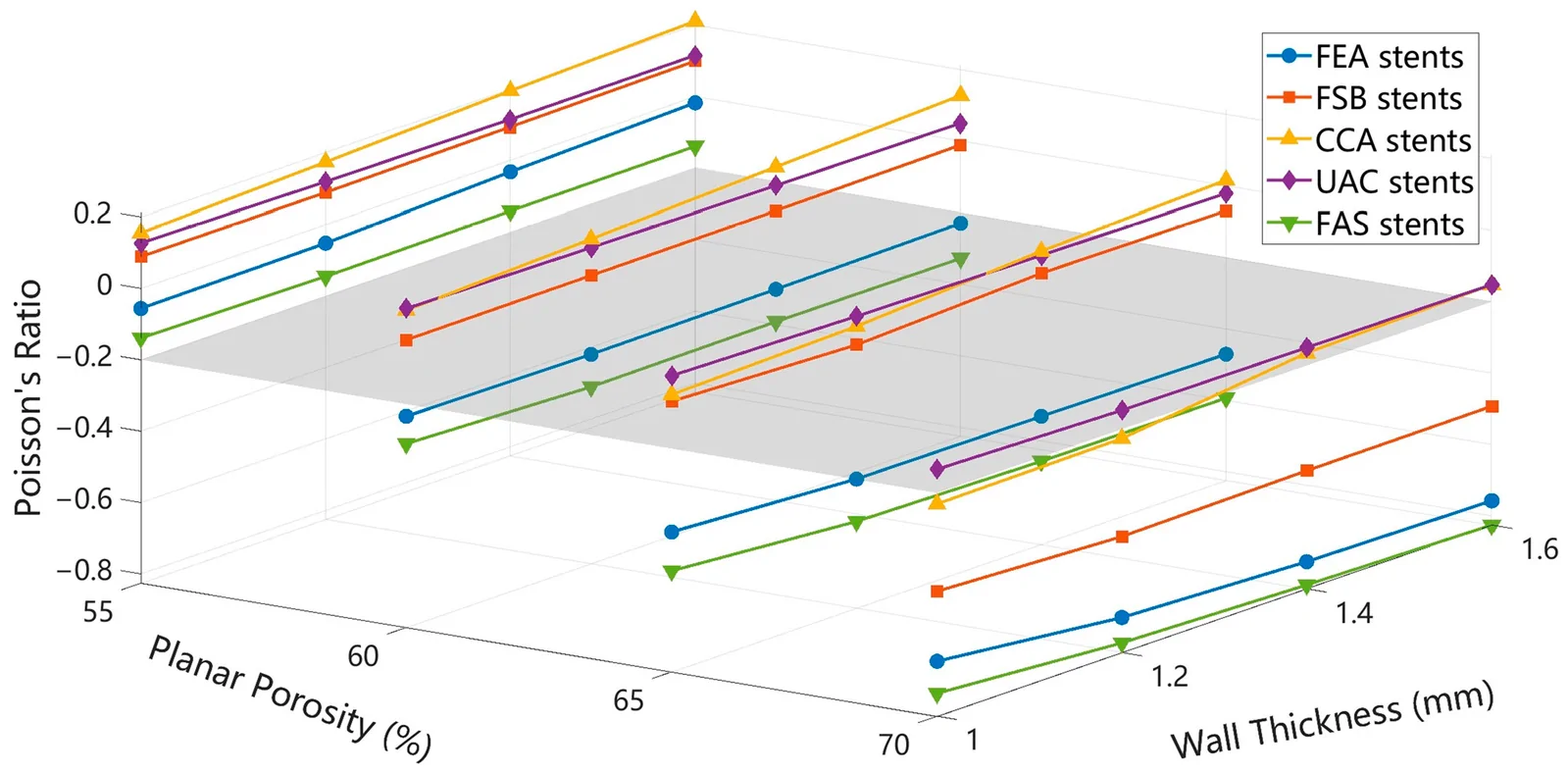
Simulation trends of Poisson’s ratio for the five stent designs under different design parameters.

When conducting radial compression simulations, the axial deformation characteristics of the stents were analyzed simultaneously. The axial displacements at both ends of the stents were extracted using the built-in probe function of the static structural module in ANSYS. The average axial deformation of the five stent configurations was systematically calculated, based on which the Poisson’s ratio of each stent was determined. The processed results were then used to generate the variation trends of Poisson’s ratio with respect to design parameters, as shown in Figure 9.

The simulation results suggest that planar porosity has a substantial influence on the Poisson’s ratio response of the investigated stent configurations. For most designs, the Poisson’s ratio tended to decrease as planar porosity increased. At a planar porosity of 55%, the majority of stents exhibited positive Poisson’s ratio values, whereas negative Poisson’s ratio behavior became more apparent at higher porosity levels, particularly at 70%. This trend may be associated with the increased deformation space available for inward rotation and re-entrant deformation of the unit cells under highly porous conditions.

Compared with planar porosity, wall thickness appears to have a relatively limited influence on the Poisson’s ratio response. A slight decrease in Poisson’s ratio is generally observed with increasing wall thickness. One possible explanation is that the cylindrical geometry of the stent results in a small increase in the effective planar porosity as wall thickness increases. However, the CCA stents exhibited a modest increasing trend in Poisson’s ratio. This behavior may be related to the outward deformation tendency of the convex curved segments during compression, which could partially offset the influence of wall-thickness-induced geometric changes.

A comparison among the FEA, FSB, and CCA stents suggests that the geometry of the transverse struts may play an important role in the auxetic response of the structure. Based on the present results, the tendency to exhibit negative Poisson’s ratio behavior appears to follow the order of flat edges, concave arcs, and convex arcs. Under radial compression, flat-edge geometries may facilitate more coordinated inward deformation of the unit cells, resulting in relatively larger transverse strains. In comparison, arc-shaped geometries tend to undergo distributed bending deformation, which may reduce the efficiency of inward displacement transfer. In addition, convex curved segments may exhibit an outward load-transfer tendency under compression, potentially weakening the auxetic response.

The comparison between the FEA and UAC stents further suggests that the symmetry of the longitudinal concave features may influence the magnitude of the negative Poisson’s ratio effect. The bidirectional concave configuration generally exhibits a stronger auxetic response than the unidirectional configuration. This observation may be associated with the more symmetric inward deformation pattern of the bidirectional topology, which could promote greater transverse contraction under equivalent loading conditions. In contrast, the presence of a relatively straight constraint side in the UAC stents may limit inward deformation, resulting in Poisson’s ratio values approaching zero or remaining slightly positive.

Furthermore, a comparison between the FSB and FAS stents indicates that geometries containing re-entrant angular features tend to exhibit a stronger negative Poisson’s ratio effect than smooth curved configurations. In the FAS stents, deformation appears to be concentrated near the re-entrant vertices, which may facilitate localized inward contraction of the unit cells. In contrast, deformation in the FSB stents is distributed over continuous curved segments, requiring bending deformation to occur over a larger region. This difference in deformation mode may contribute to the relatively weaker auxetic response observed in the FSB topology.

### 3.4. Validation of Numerical Simulations and Analysis of Fabrication Deviations

#### 3.4.1. Experimental Characterization of Stent Mechanical Response

**Figure 10:**
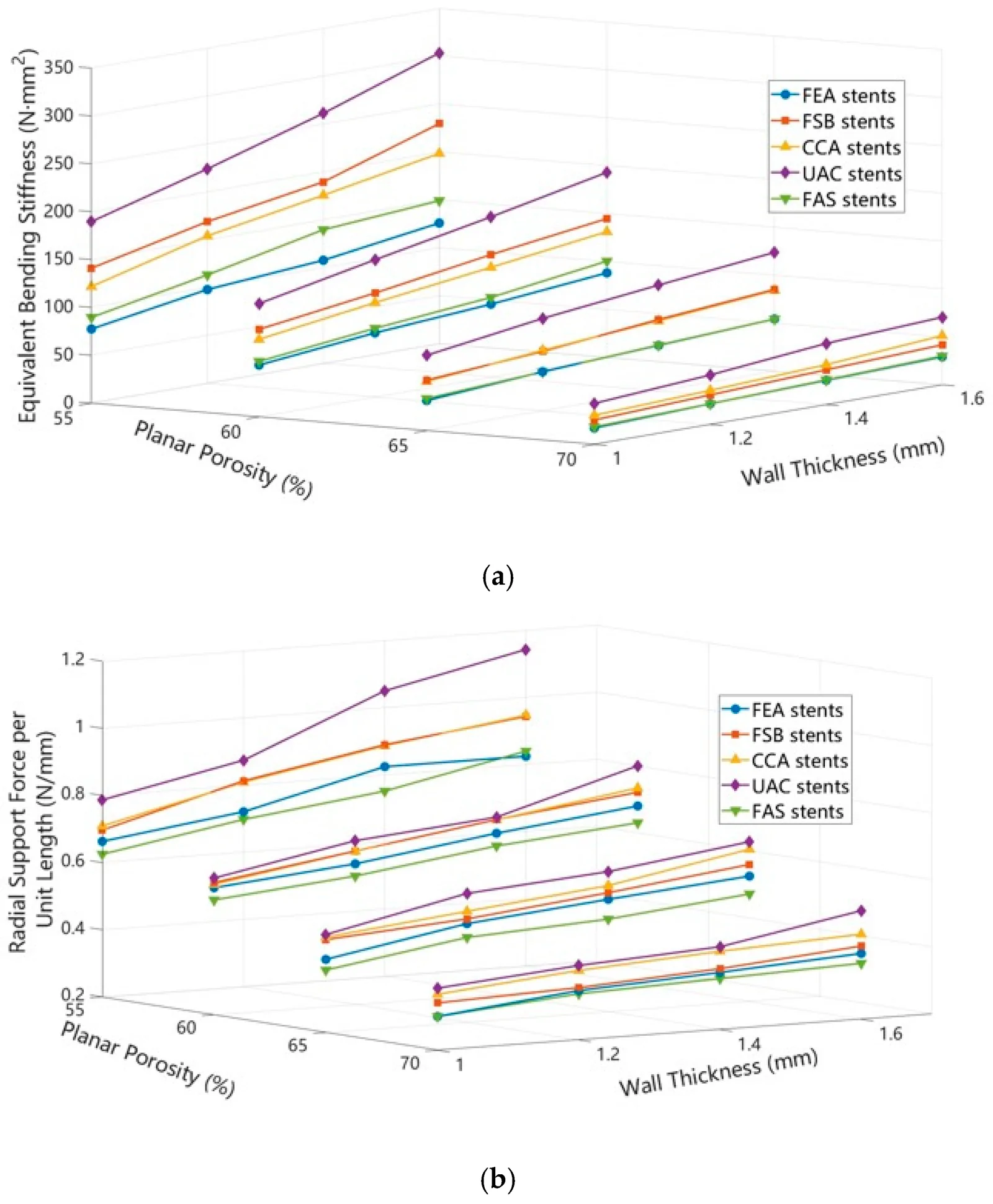
(**a**) Experimental measurements of equivalent bending stiffness for the five stent designs under different design parameters; (**b**) experimental measurements of radial support force per unit length for the five stent designs under different design parameters;.

To validate the reliability of the finite element models in predicting the mechanical behavior of polymer stents, axial cantilever bending tests and radial compression tests were conducted on 80 thermoplastic polyurethane stent specimens fabricated by fused deposition modeling, covering different combinations of the design parameters. In the axial bending tests, each specimen group was tested in triplicate, and the average reaction force was adopted to calculate the equivalent bending stiffness. The resulting trends in equivalent bending stiffness for the five stent configurations under different planar porosities are presented in Figure 10a. In the radial compression tests, each specimen was tested three times to obtain the average reaction force. The radial support force was then normalized by the actual length of the stent to acquire the radial support force per unit length. The corresponding variation trends for the five stent configurations under different planar porosities are shown in Figure 10b.

The experimental results show that both the axial equivalent bending stiffness and the radial support force per unit length generally increased with increasing wall thickness and decreased with increasing planar porosity across all topological configurations. These observations are consistent with the expected strengthening effect of wall thickness on the structural section modulus and the reduction in material continuity associated with higher porosity levels. Under identical parameter combinations, the experimentally measured performance rankings were in good agreement with the numerical predictions. In terms of axial flexibility, the FEA stents exhibited the lowest bending stiffness among the investigated designs. Regarding radial support performance, the UAC stents generally demonstrated the highest support capability, whereas the FAS stents exhibited comparatively lower radial stiffness. This behavior may be associated with the presence of sharp re-entrant corners, which tend to promote localized stress concentration and influence the overall load-bearing response of the structure.

#### 3.4.2. Error Analysis

**Figure 11:**
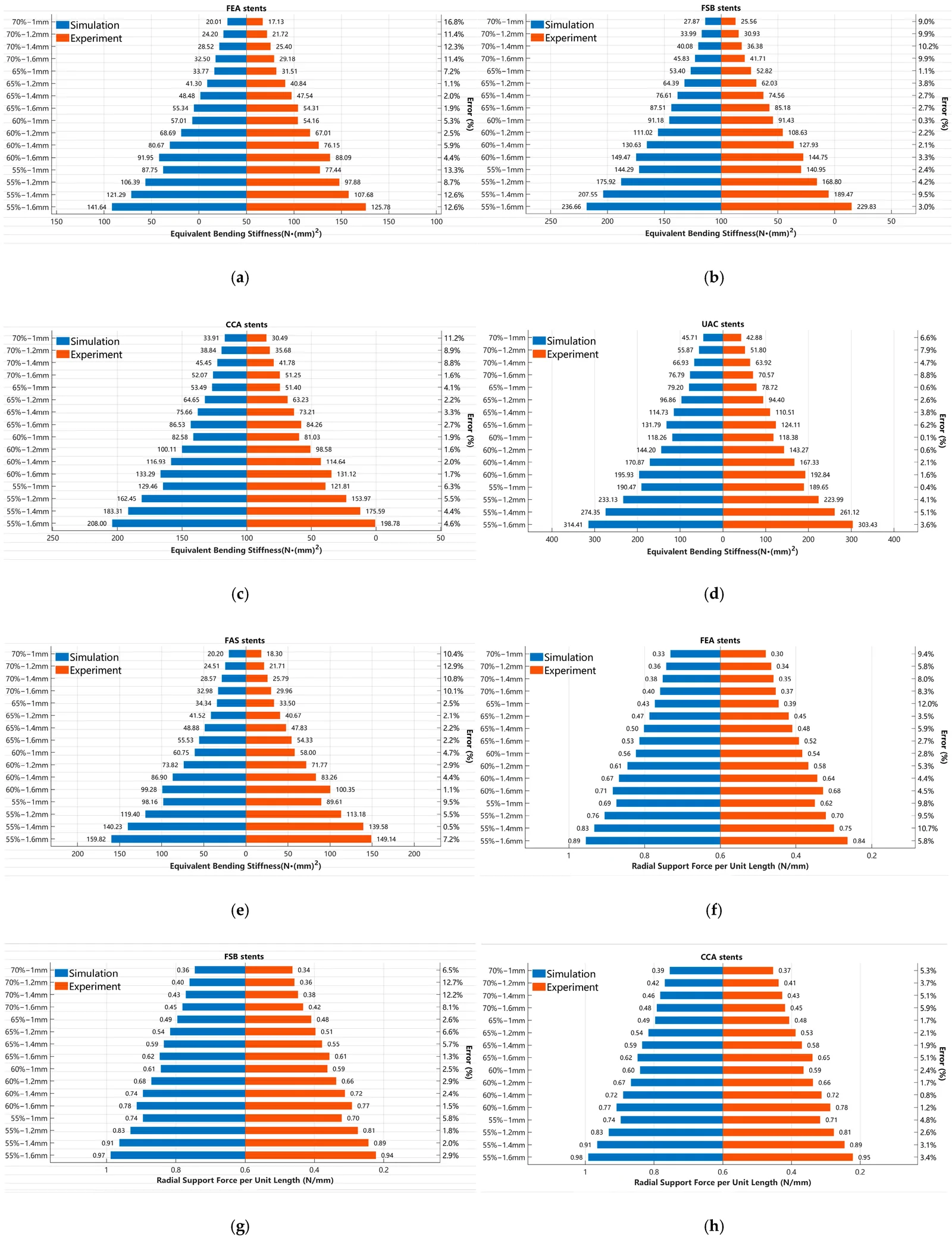

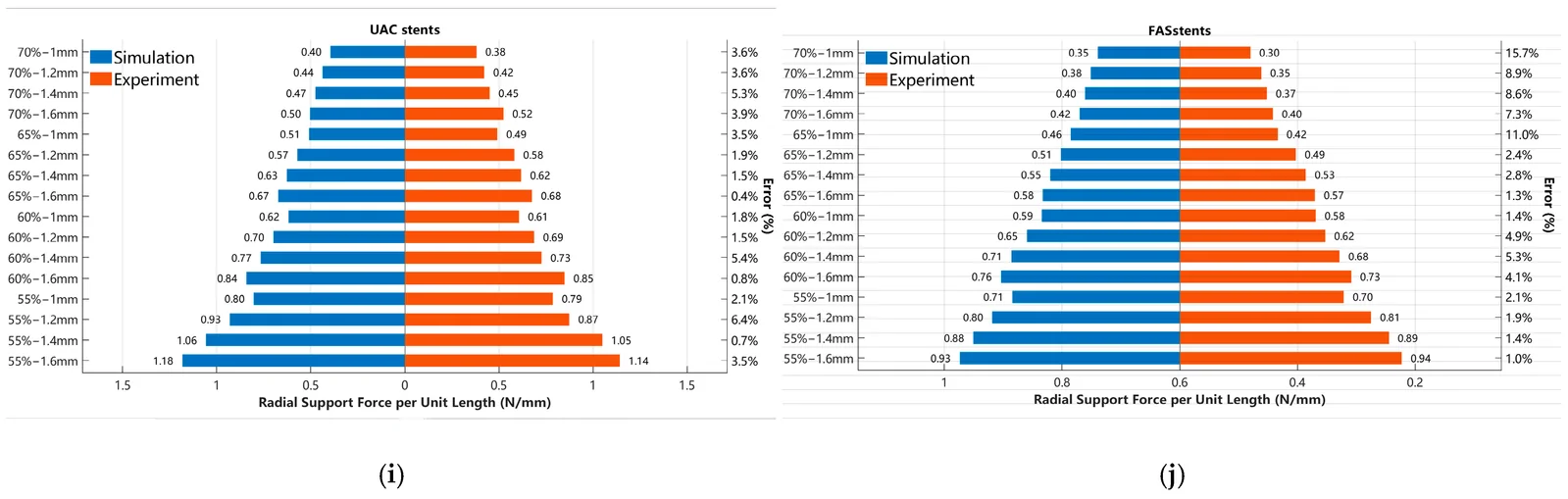
Comparison between the simulation and experimental results of axial bending and radial compression for the five stent configurations. The blue regions represent simulation results, whereas the red regions represent experimental results: (**a**) FEA stent under axial bending; (**b**) FSB stent under axial bending; (**c**) CCA stent under axial bending; (**d**) UAC stent under axial bending; (**e**) FAS stent under axial bending; (**f**) FEA stent under radial compression; (**g**) FSB stent under radial compression; (**h**) CCA stent under radial compression; (**i**) UAC stent under radial compression; (**j**) FAS stent under radial compression.

To provide a more intuitive comparison between the simulation and experimental results, this study systematically compiled the performance of the stents under various design parameters in both the axial bending and radial compression experiments, as illustrated in Figure 11a–j. In these figures, the blue regions represent simulation data, while the red regions denote experimental measurements.

To quantitatively assess the deviation between the simulation and experiment, the relative error δ was introduced. The relative error was calculated by substituting the simulation and experimental values into Equation (1):

(1) $$\delta =\frac{V_{Sim}-V_{exp}}{V_{exp}}\;\times \;100\%$$

where $V_{Sim}$ represents the value obtained from finite element simulation, and $V_{exp}$ denotes the experimentally measured value.

Quantitative analysis showed that among the 80 specimens evaluated in the axial bending tests, 16.25% exhibited relative errors greater than 10%, with a maximum error of 16.80%. These results suggest that the cantilever beam bending model can provide a reasonable prediction of stent flexibility. In the radial compression tests, only 6.25% of the 80 specimens exhibited relative errors exceeding 10%, with a maximum error of 15.70%. This finding indicates that the uniform centripetal loading model established in the cylindrical coordinate system is capable of capturing the radial support behavior of the stents with acceptable accuracy.

Although the simulation predictions and experimental measurements exhibited generally consistent variation trends, quantitative comparisons revealed that the experimentally measured mechanical responses were, in most cases, slightly lower than the corresponding finite element predictions. This systematic discrepancy may be associated with several factors related to both the manufacturing process and the assumptions adopted in the numerical model.

First, geometric deviations introduced during the fused deposition modeling process are likely to be an important contributing factor. Although the Raise3D E2 printing platform provides a manufacturing accuracy of approximately 0.05–0.10 mm, such deviations become significant for slender struts with a wall thickness of only 1.0 mm, potentially altering the cross-sectional moment of inertia and consequently affecting the measured stiffness. This effect appears to be more pronounced in stents with a planar porosity of 70%, where the thinner strut geometry is more sensitive to dimensional variations. Second, the finite element model assumes thermoplastic polyurethane to be an isotropic linear elastic material. However, the layer-by-layer deposition inherent to fused deposition modeling can introduce direction-dependent mechanical behavior in the fabricated specimens. The deposited filaments may exhibit different load-transfer characteristics along the printing direction and across adjacent layers because of differences in filament orientation and interlayer bonding, resulting in variations in effective stiffness and deformation response. Consequently, the mechanical response of the printed TPU structures may show a certain degree of deviation from that predicted using an ideal isotropic material model, particularly when the structural deformation involves both in-plane filament deformation and interlayer interaction [57,58]. In addition, frictional interactions present in the experimental boundary conditions may also contribute to the observed discrepancies. Physical contact between the stent and the simulated vessel components or fixation fixtures was simplified as ideal constraints in the numerical model. As a result, the experimentally measured reaction forces may deviate slightly from the theoretical predictions.

### 3.5. Integrated Mechanical Performance and Coordinated Tunability of Five Auxetic Stent Structures

**Figure 12:**
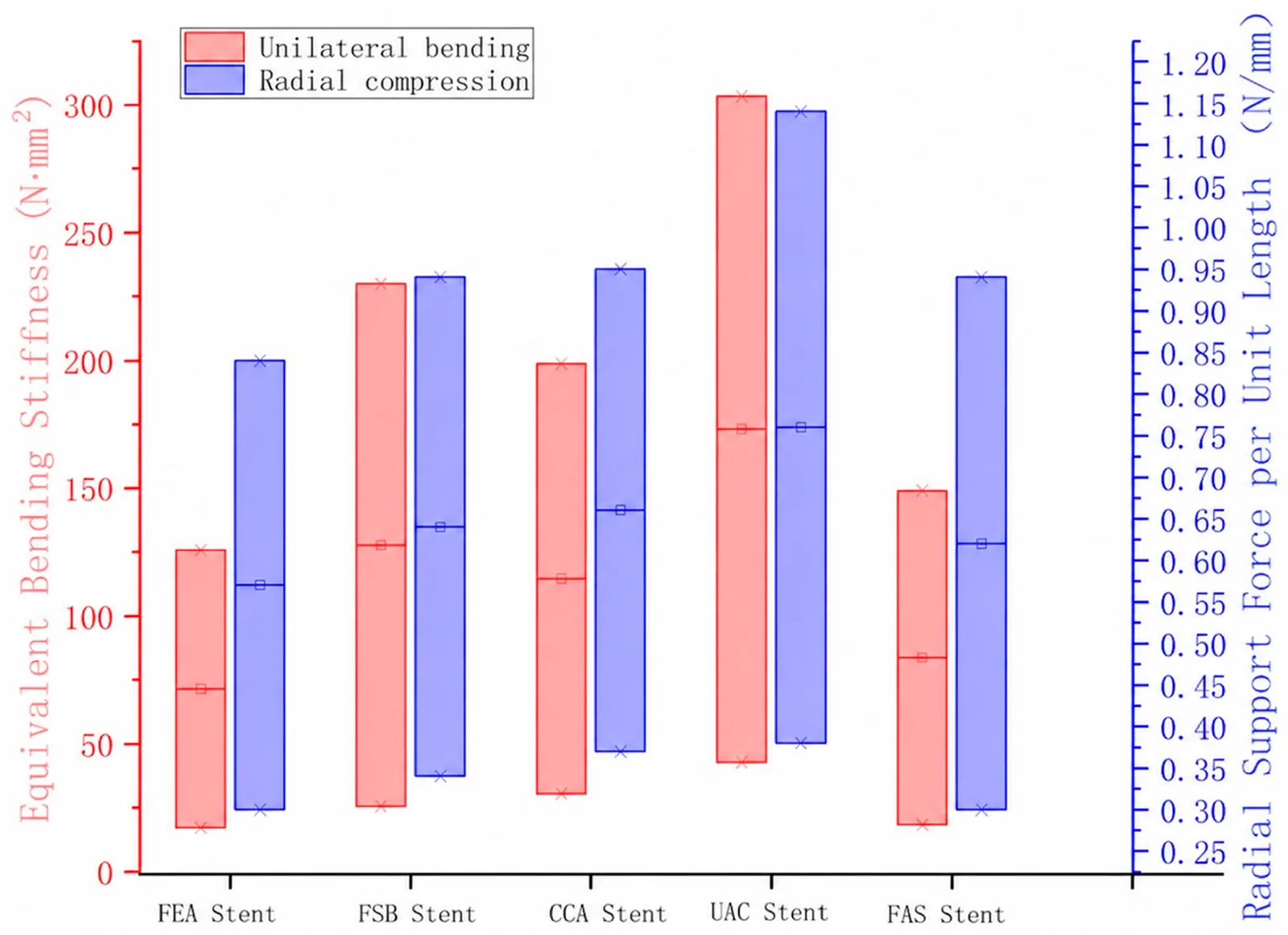
Mechanical performance ranges of the five TPU stent structures in terms of equivalent bending stiffness and radial support force per unit length.

Figure 12 summarizes the ranges of equivalent bending stiffness and radial support force per unit length for the five auxetic stent structures under different combinations of planar porosity and wall thickness. The results indicate that different topologies first establish distinct baseline mechanical tendencies, while variations in geometric parameters further expand their respective tunable performance ranges. The FEA structure exhibited an equivalent bending stiffness of 17.13–125.78 N·mm^2^ and a radial support force per unit length of 0.30–0.84 N/mm. The relatively narrow variation ranges of both metrics indicate that its overall mechanical response is biased toward maintaining higher bending flexibility. In contrast, the UAC structure showed the broadest tunable performance range, with the equivalent bending stiffness varying from 42.88 to 303.43 N·mm^2^ and the radial support force increasing from 0.38 to 1.14 N/mm, demonstrating greater mechanical tunability in response to changes in planar porosity and wall thickness. The FSB and CCA structures were mainly distributed between the FEA and UAC structures, exhibiting intermediate performance ranges that provided a relatively balanced combination of bending compliance and radial support. The FAS structure exhibited an equivalent bending stiffness of 18.30–149.14 N·mm^2^, while its radial support force could be tuned within the range of 0.30–0.94 N/mm, indicating a relatively broad radial support adjustment range under low-to-moderate bending stiffness.

## 4. Conclusions

This study systematically investigated the mechanical characteristics of five polymeric topological stent configurations fabricated using fused deposition modeling technology. The results provide insights into the coupled influences of topology, planar porosity, and wall thickness on stent flexibility, radial support performance, and Poisson’s ratio behavior. Through the combined analysis of numerical simulations and experimental validation, geometric curvature and structural symmetry were identified as important factors influencing the mechanical responses of the stents. The introduction of continuous curved geometries appeared to enhance radial stiffness through an arch-like load transfer mechanism while promoting a more uniform stress distribution. In contrast, the introduction of unilateral geometric constraints tended to improve radial support performance at the expense of a certain degree of axial flexibility. These observations may provide useful design guidance for tailoring the mechanical performance of polymer stents through microstructural geometric modification.

Based on the obtained results, a hierarchical parameter design strategy for FDM-printed elastomeric stent structures is proposed as follows:

(1) Topology selection as the primary determinant of axial dimensional stability

Among the investigated design parameters, the Poisson’s ratio response exhibited the highest sensitivity to topological configuration. For applications where minimizing axial shortening is desirable, topology selection may therefore represent an important design consideration. The present results suggest that structures containing sharp corners or straight hinge-like features generally exhibit stronger auxetic behavior than fully smooth curved designs. In particular, the UAC stent maintained a Poisson’s ratio close to zero even at a planar porosity of 70%, indicating a relatively high resistance to axial contraction. These findings suggest that topological configuration plays a critical role in governing axial dimensional stability.

(2) Planar porosity as the primary macroscopic tuning parameter

After the topological architecture has been determined, planar porosity may serve as an effective parameter for adjusting overall mechanical performance. The results indicate that planar porosity substantially influences the transition between truss-dominated and shell-dominated deformation behaviors. Taking the UAC stent as an example, when the wall thickness was fixed at 1.6 mm, reducing the planar porosity from 70% to 55% increased the radial support force per unit length from 0.52 N/mm to 1.14 N/mm. This enhancement in radial support was accompanied by a considerable increase in equivalent bending stiffness from 70.57 N·mm^2^ to 303.43 N·mm^2^, reflecting a corresponding reduction in flexibility. Such observations suggest that planar porosity may function as an effective coarse-tuning parameter for rapidly shifting stent performance toward different mechanical targets. Furthermore, stress analysis under radial compression showed that peak equivalent stress was relatively insensitive to changes in planar porosity. Under the above loading conditions, the maximum stress varied only modestly between 0.960 MPa and 1.082 MPa.

(3) Wall thickness as a fine-tuning parameter for mechanical optimization

Compared with planar porosity, wall thickness primarily induced gradual changes in mechanical performance and may therefore be more suitable for fine adjustment. Experimental results revealed approximately linear positive relationships between wall thickness and key mechanical indicators, although the magnitude of these effects was generally smaller than that associated with planar porosity. For example, at a planar porosity of 55%, increasing the wall thickness from 1.0 mm to 1.6 mm increased the radial support force per unit length of the UAC stent from 0.79 N/mm to 1.14 N/mm, while the equivalent bending stiffness increased from 189.65 N·mm^2^ to 303.43 N·mm^2^. These changes suggest a relatively moderate influence on flexibility compared with porosity adjustment. Nevertheless, wall thickness exhibited a more pronounced effect on stress concentration. At a planar porosity of 70%, increasing the wall thickness by only 0.6 mm resulted in an increase in peak equivalent stress from 0.821 MPa to 1.082 MPa. This sensitivity suggests that wall thickness should be adjusted with the consideration of potential stress amplification effects when optimizing stent performance.

Although the newly developed auxetic stents demonstrate the potential to balance radial support, bending flexibility, and axial contraction through topological and geometric optimization, the present study remains a proof-of-concept investigation at the macroscopic mechanical level. Several challenges related to clinical translation and manufacturing therefore warrant further study.

First, limited by the manufacturing resolution of conventional fused deposition modeling (FDM), existing prototypes have thick walls and insufficient surface microstructural accuracy. These macroscopic geometric features can alter the local hemodynamic environment in vascular applications, disturbing flow patterns and raising risks of adverse biological responses. Accordingly, future translational research should adopt higher-resolution additive manufacturing methods, including digital light processing-based stereolithography and other microfabrication techniques, to produce finer structures. In addition, the in vivo hemodynamic behavior of such porous flexible tubular topologies involves complex fluid–structure interaction. Further investigations via computational fluid dynamics simulations and microfluidic experiments are required to clarify how local planar porosity, nodal constraints and geometric gradients affect vascular wall shear stress and flow characteristics.

Second, the inherent radiolucency of TPU limits its visibility under clinical imaging. Future research may therefore focus on radiopaque polymer composites by incorporating biocompatible contrast agents, such as barium sulfate (BaSO_4_), gold (Au), or tantalum (Ta), to enhance radiographic visibility while preserving mechanical performance.

Finally, the long-term biomechanical reliability of TPU stents remains to be validated. Owing to the viscoelastic nature of TPU, future work should systematically investigate fatigue behavior, cyclic hysteresis, creep, and long-term degradation under simulated physiological conditions to further evaluate their durability and clinical potential.

## Figures and Tables

**Table 1:** Basic mechanical indicators of TPU.

Property	Density	100% Modulus	Tensile Strength	Elongation at Break
Testing method	[47,48]	[49,50]	[49,50]	[49,50]
Typical value	1.12 g/cm^3^	3.40 ± 0.42 (MPa)	12.1 ± 0.82 (MPa)	638.8 ± 15.3 (%)

## Data Availability

All data are available from the corresponding author (DS) upon reasonable request.

## Supplementary Materials

The following supporting information can be downloaded at: https://www.mdpi.com/article/10.3390/mi17091092/s1, Figure S1: Front view of printed stent specimens for the five designs under different planar porosity and wall thickness combinations. From left to right and top to bottom, wall thicknesses are 1.0 mm, 1.2 mm, 1.4 mm, and 1.6 mm; Figure S2: Top view of printed stent specimens for the five designs under different planar porosity and wall thickness combinations. From left to right and top to bottom, wall thicknesses are 1.0 mm, 1.2 mm, 1.4 mm, and 1.6 mm; Figure S3: Single-side bending simulation equivalent stress of the Flat-Edge and Arc Composite Concave Stent under different planar porosity and wall thickness combinations. From left to right and top to bottom, wall thicknesses are 1.0 mm, 1.2 mm, 1.4 mm, and 1.6 mm; Figure S4: Single-side bending simulation equivalent stress of the Fully Smooth Bidirectional Concave Stent under different planar porosity and wall thickness combinations. From left to right and top to bottom, wall thicknesses are 1.0 mm, 1.2 mm, 1.4 mm, and 1.6 mm; Figure S5: Single-side bending simulation equivalent stress of the Concave-Convex Arc Composite Stent under different planar porosity and wall thickness combinations. From left to right and top to bottom, wall thicknesses are 1.0 mm, 1.2 mm, 1.4 mm, and 1.6 mm; Figure S6: Single-side bending simulation equivalent stress of the Unidirectional Arc Concave Stent under different planar porosity and wall thickness combinations. From left to right and top to bottom, wall thicknesses are 1.0 mm, 1.2 mm, 1.4 mm, and 1.6 mm; Figure S7: Single-side bending simulation equivalent stress of the Four-Angled Star-Shaped Stent under different planar porosity and wall thickness combinations. From left to right and top to bottom, wall thicknesses are 1.0 mm, 1.2 mm, 1.4 mm, and 1.6 mm; Figure S8: Equivalent stress distribution under radial compression simulation of the Flat-Edge and Arc Composite Concave Stent with varying planar porosity and wall thickness. From left to right and top to bottom, the wall thicknesses are 1.0 mm, 1.2 mm, 1.4 mm, and 1.6 mm; Figure S9: Equivalent stress distribution under radial compression simulation of the Fully Smooth Bidirectional Concave Stent with varying planar porosity and wall thickness. From left to right and top to bottom, the wall thicknesses are 1.0 mm, 1.2 mm, 1.4 mm, and 1.6 mm; Figure S10: Equivalent stress distribution under radial compression simulation of the Concave-Convex Arc Composite Stent with varying planar porosity and wall thickness. From left to right and top to bottom, the wall thicknesses are 1.0 mm, 1.2 mm, 1.4 mm, and 1.6 mm; Figure S11: Equivalent stress distribution under radial compression simulation of the Unidirectional Arc Concave Stent with varying planar porosity and wall thickness. From left to right and top to bottom, the wall thicknesses are 1.0 mm, 1.2 mm, 1.4 mm, and 1.6 mm; Figure S12: Equivalent stress distribution under radial compression simulation of the Four-Angled Star-Shaped Stent with varying planar porosity and wall thickness. From left to right and top to bottom, the wall thicknesses are 1.0 mm, 1.2 mm, 1.4 mm, and 1.6 mm.
