# Supplementary material for: Topology-Driven Mechanical Tuning of FDM-Printed TPU Tubular Lattices with Auxetic Architectures
Source: Micromachines (Basel). 2026 Sep 17;17(9):1092. doi: 10.3390/mi17091092 (PMC13609363; doi:10.3390/mi17091092)
Supplement: Supplementary file 1 [file micromachines-17-01092-s001.zip › micromachines-4535524-supplementary.pdf]

Supplementary material

Figure.S1 presents the macroscopic front-view images of five stent unit structures fabricated using a Raise3D printer with white thermoplastic polyurethane filaments (material properties are listed in Table 1). The figure aims to visually illustrate the differences in planar porosity and structural design among the stents.

Taking **Figure.S1**(a) as an example, all stents exhibit a planar porosity of 55%. The specimens are arranged in four rows corresponding to different wall thicknesses: the upper-left group represents 1.0 mm, the upper-right 1.2 mm, the lower-left 1.4 mm, and the lower-right 1.6 mm. Within each row, the five stent structures from left to right are: flat-edge–arc concave composite, fully smooth bidirectional concave, concave–convex arc composite, unidirectional arc concave, and four-point star-shaped structures.

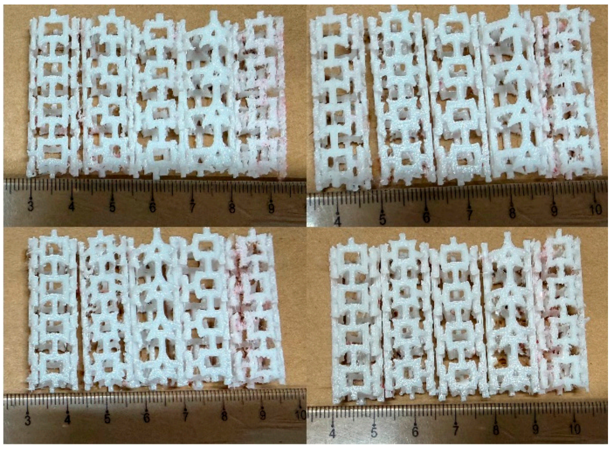

Planar Porosity 55%

(a)

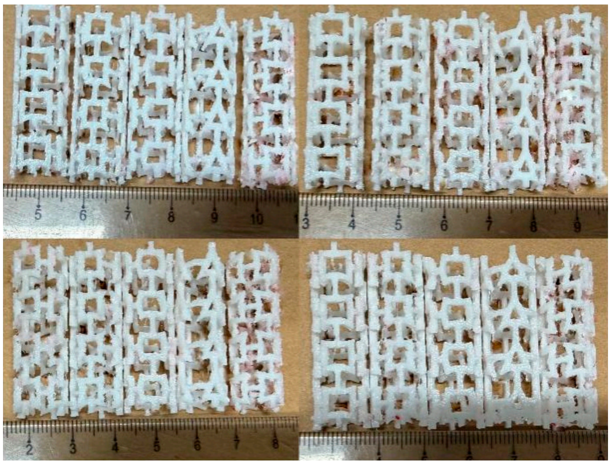

Planar Porosity 60%

(b)

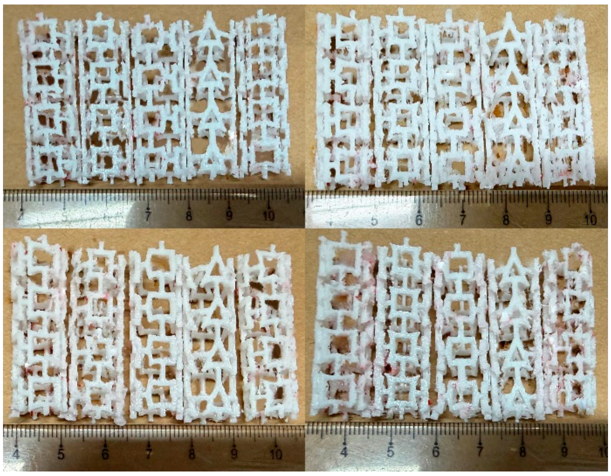

Planar Porosity 65%

(c)

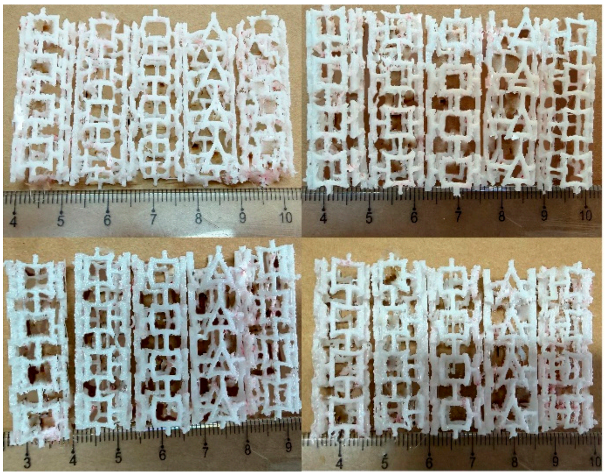

Planar Porosity 70%

(d)

Figure.S1 Front view of printed stent specimens for the five designs under different planar porosity and wall thickness combinations. From left to right and top to bottom, wall thicknesses are 1.0 mm, 1.2 mm, 1.4 mm, and 1.6 mm

Figure.S2 presents the macroscopic top-view images of five stent unit structures fabricated using a Raise3D printer with white thermoplastic polyurethane filaments (material properties are listed in Table 1). The figure aims to visually demonstrate the variation in wall thickness among different stent designs. Taking **Figure.S2(a)** as an example, all stents exhibit a planar porosity of 55%. The specimens are arranged in four rows corresponding to different wall thicknesses: the upper-left group represents 1.0 mm, the upper-right 1.2 mm, the lower-left 1.4 mm, and the lower-right 1.6 mm. Within each row, the five stent structures from left to right are: flat-edge–arc concave composite, fully smooth bidirectional concave, concave–convex arc composite, unidirectional arc concave, and four-point star-shaped structures.

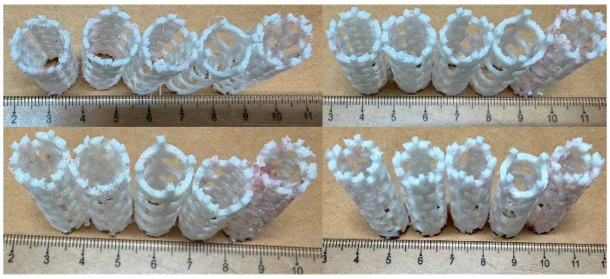

Planar Porosity 55%

(a)

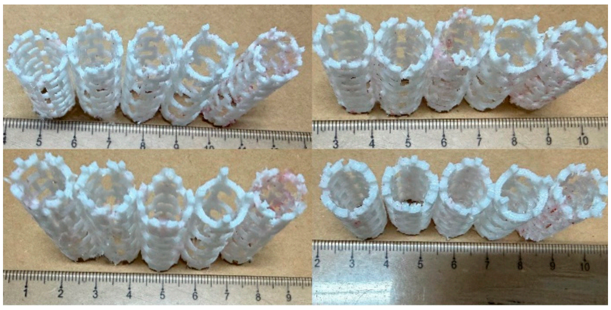

Planar Porosity 60%

(b)

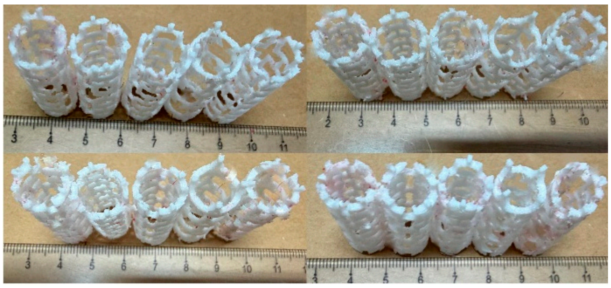

Planar Porosity 65%

(c)

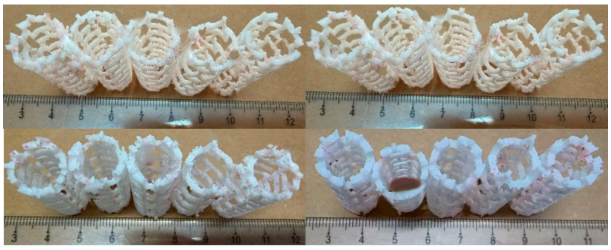

Planar Porosity 70%

(d)

Figure.S2 Top view of printed stent specimens for the five designs under different planar porosity and wall thickness combinations. From left to right and top to bottom, wall thicknesses are 1.0 mm, 1.2 mm, 1.4 mm, and 1.6 mm

Figures.S3-S7 present the simulation results of unilateral bending for five stent designs under different combinations of planar porosity and wall thickness. The detailed simulation procedures are described in the Materials and Methods section.

Taking **Figure.S3** as an example, **Figure.S3**(a) corresponds to the flat-edge–arc concave composite stent with a planar porosity of 70%. The four quadrants represent different wall thicknesses: 1.0 mm (upper left), 1.2 mm (upper right), 1.4 mm (lower left), and 1.6 mm (lower right).

Similarly, **Figures.S3(b), (c), and (d)** correspond to planar porosities of 65%, 60%, and 55%, respectively, with the same wall thickness distribution as in **Figure.S3**(a).

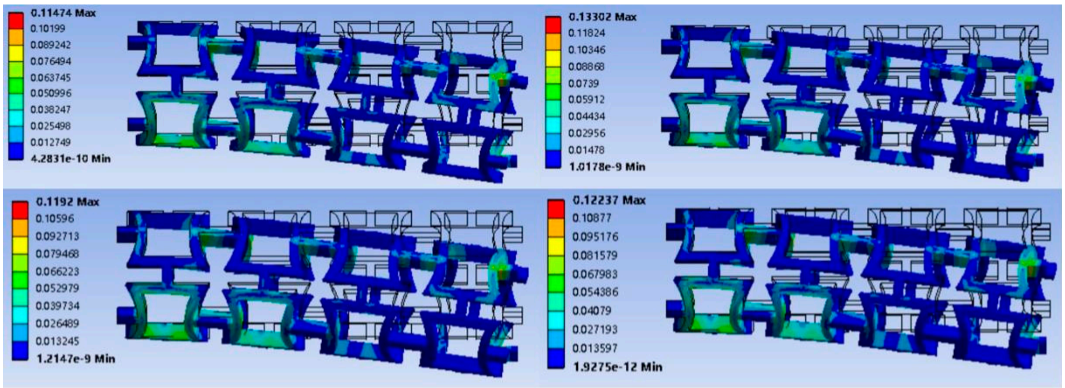

Planar Porosity 70%

(a)

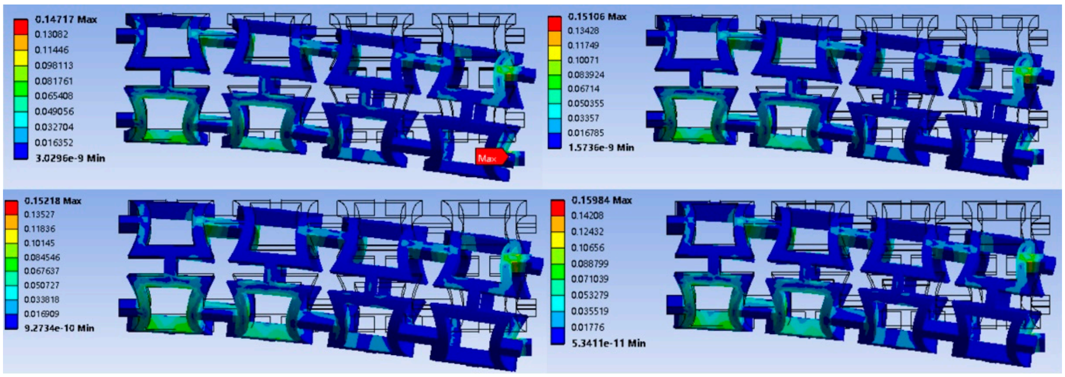

Planar Porosity 65%

(b)

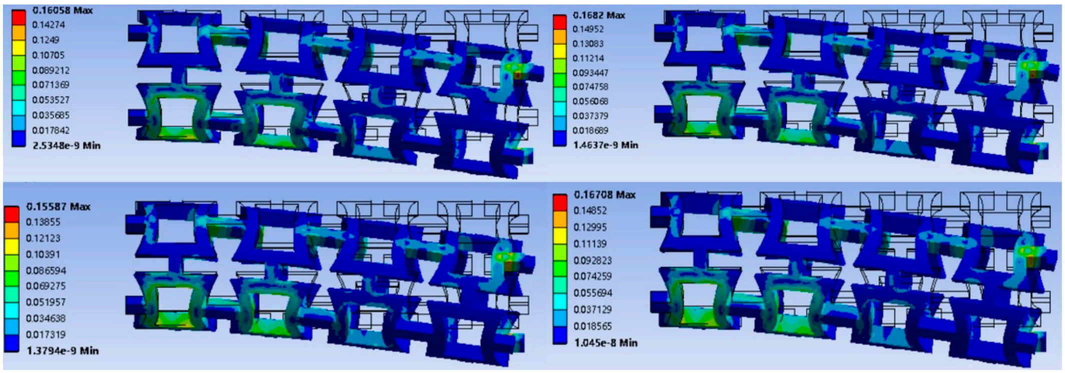

Planar Porosity 60%

(c)

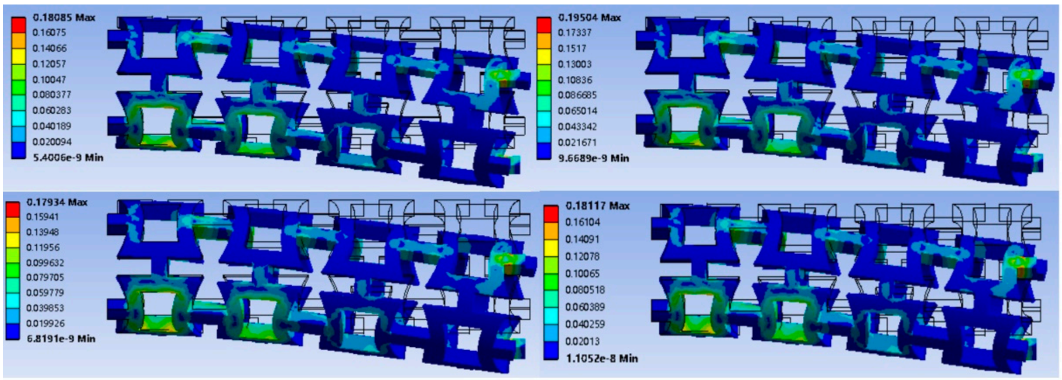

Planar Porosity 55%

(d)

Figure.S3 Single-side bending simulation equivalent stress of the **Flat-Edge and Arc Composite Concave Stent** under different planar porosity and wall thickness combinations. From left to right and top to bottom, wall thicknesses are 1.0 mm, 1.2 mm, 1.4 mm, and 1.6 mm

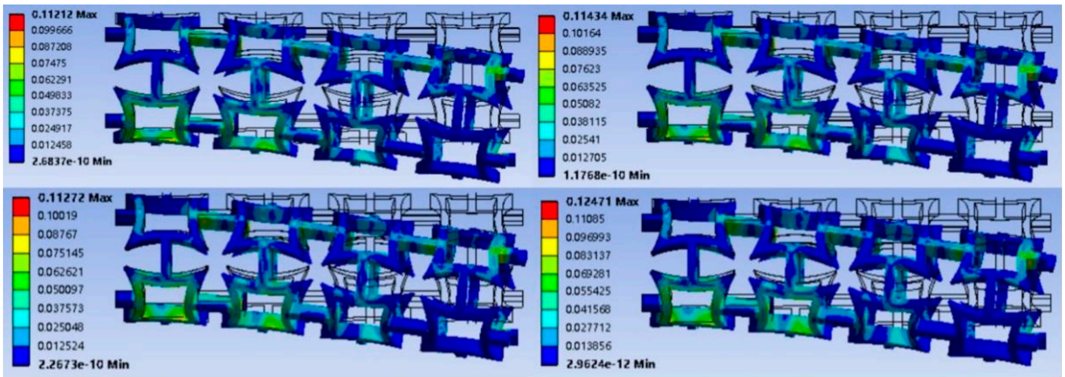

Planar Porosity 70%

(a)

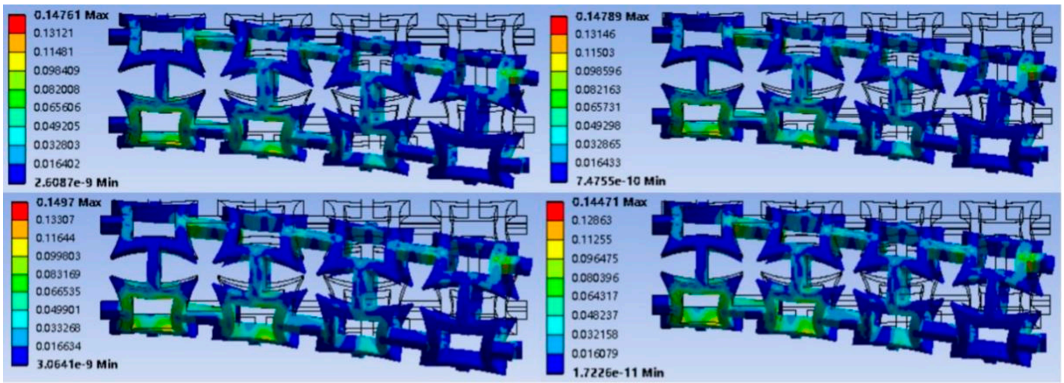

Planar Porosity 65%

(b)

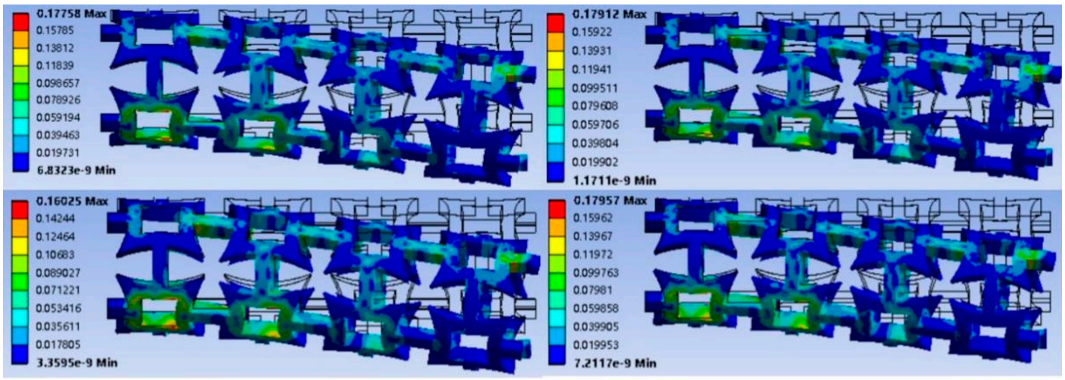

Planar Porosity 60%

(c)

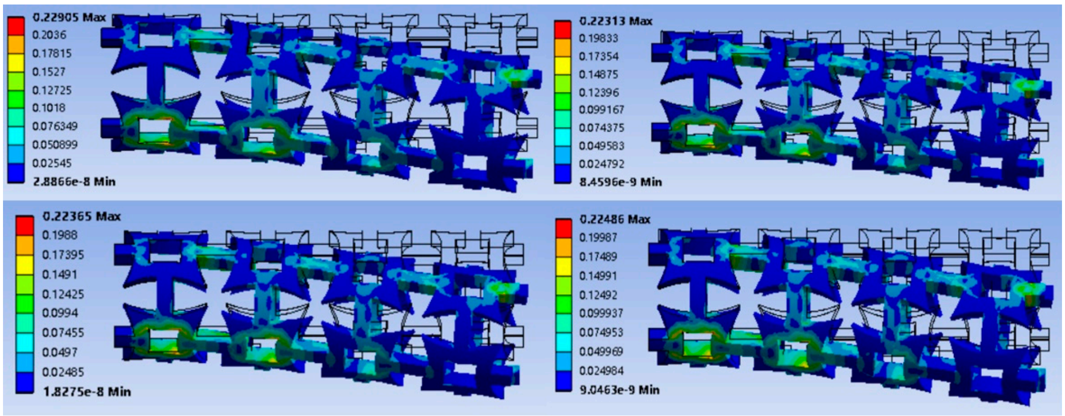

Planar Porosity 55%

(d)

Figure.S4 Single-side bending simulation equivalent stress of the Fully Smooth Bidirectional Concave Stent under different planar porosity and wall thickness combinations. From left to right and top to bottom, wall thicknesses are 1.0 mm, 1.2 mm, 1.4 mm, and 1.6 mm

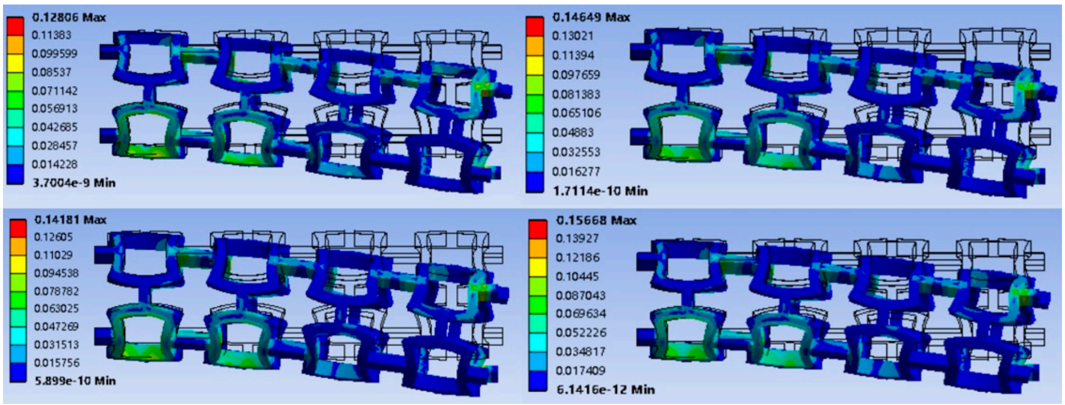

Planar Porosity 70%

(a)

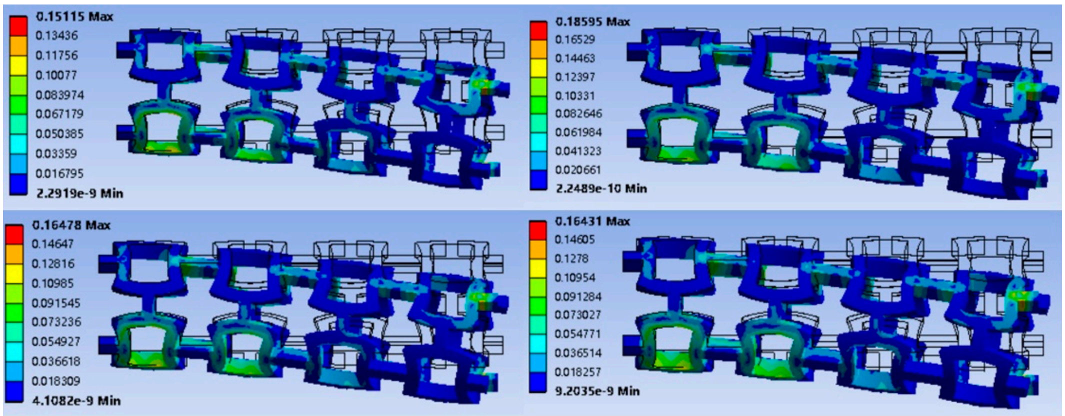

Planar Porosity 65%

(b)

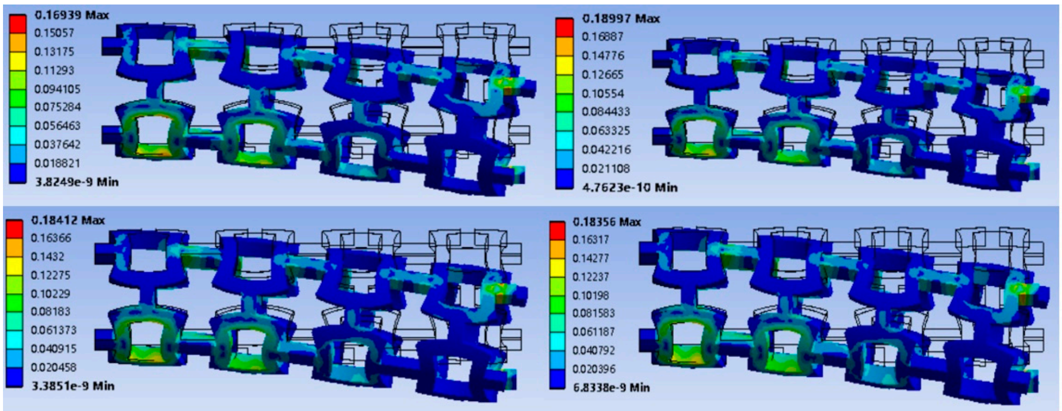

Planar Porosity 60%

(c)

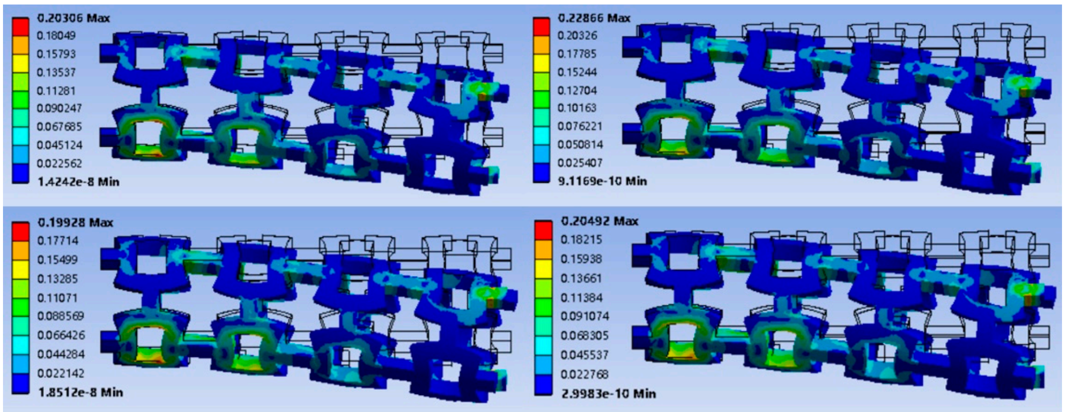

Planar Porosity 55%

(d)

Figure.S5 Single-side bending simulation equivalent stress of the Concave-Convex Arc Composite Stent under different planar porosity and wall thickness combinations. From left to right and top to bottom, wall thicknesses are 1.0 mm, 1.2 mm, 1.4 mm, and 1.6 mm

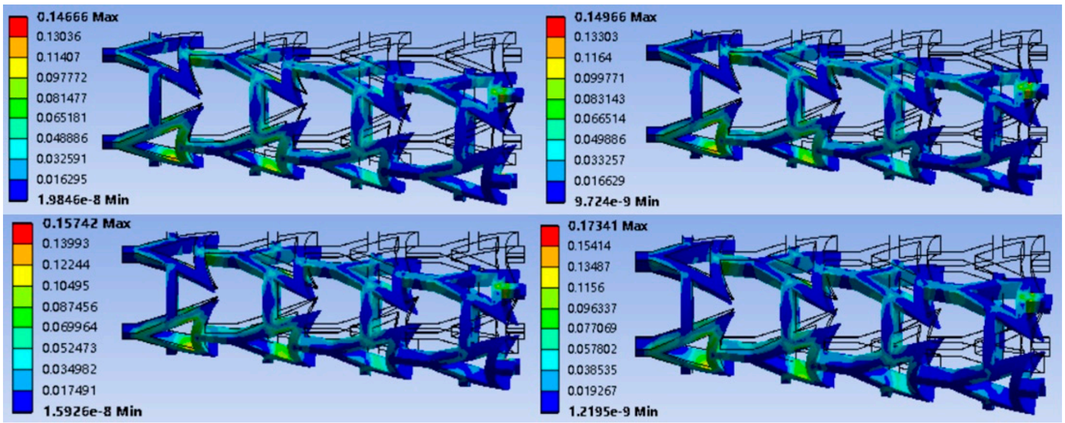

Planar Porosity 70%

(a)

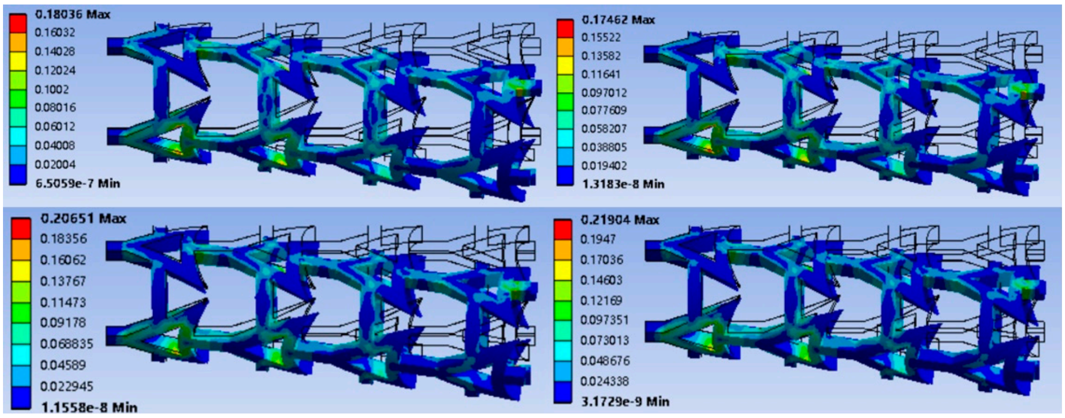

Planar Porosity 65%

(b)

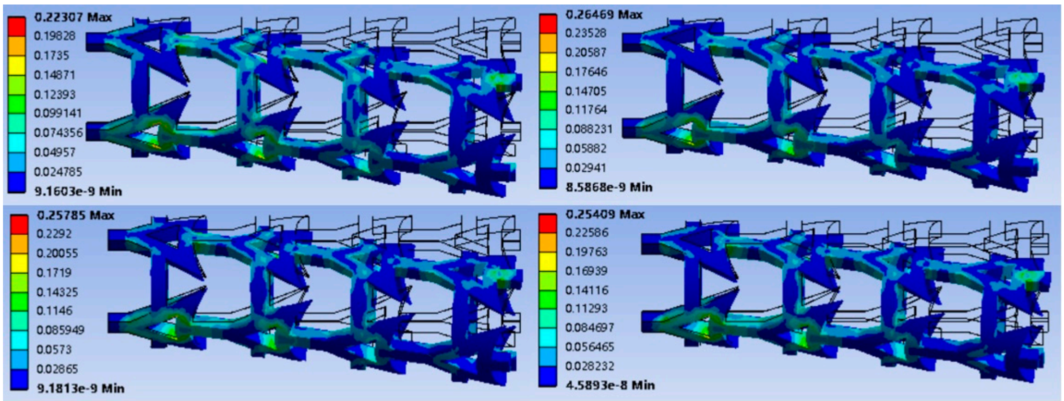

Planar Porosity 60%

(c)

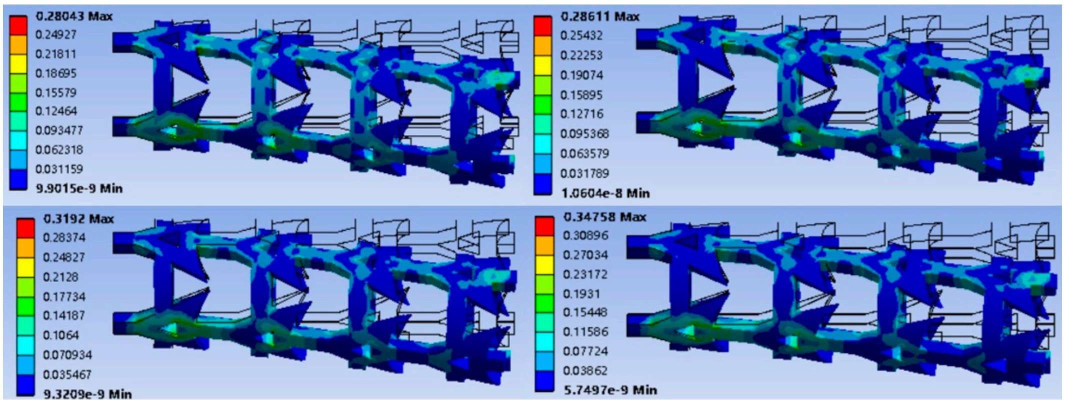

Planar Porosity 55%

(d)

Figure.S6 Single-side bending simulation equivalent stress of the Unidirectional Arc Concave Stent under different planar porosity and wall thickness combinations. From left to right and top to bottom, wall thicknesses are 1.0 mm, 1.2 mm, 1.4 mm, and 1.6 mm

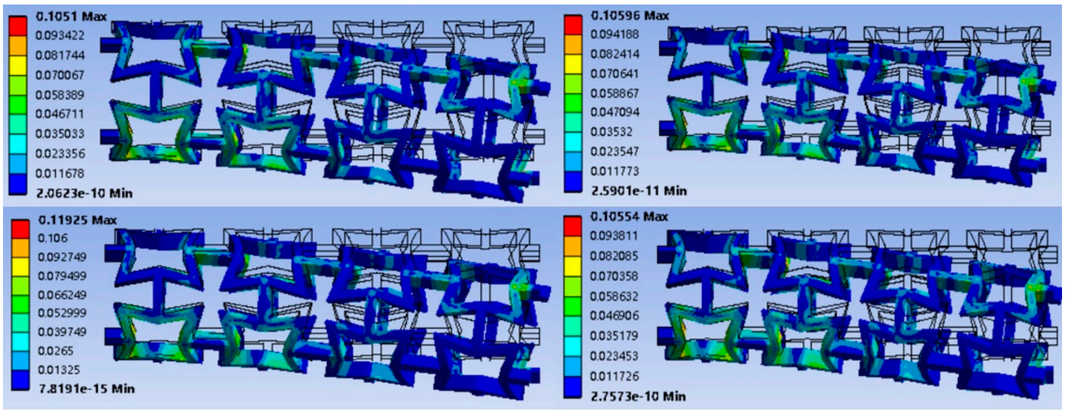

Planar Porosity 70%

(a)

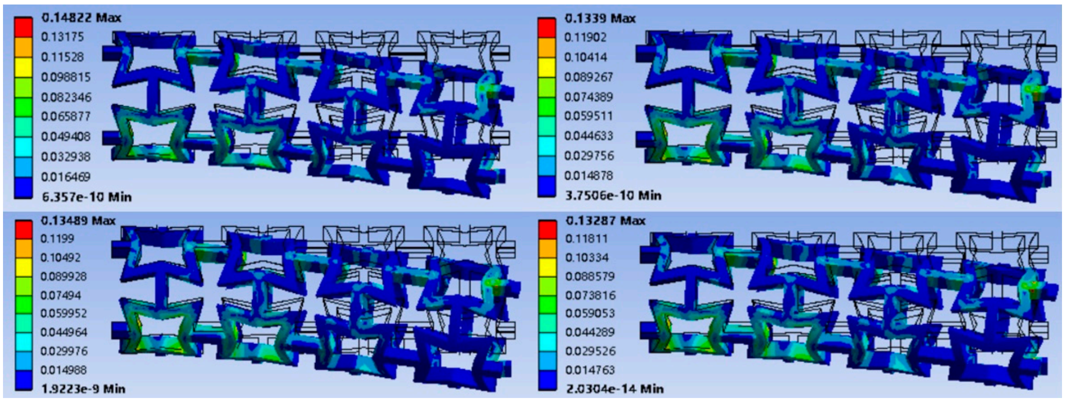

Planar Porosity 65%

(b)

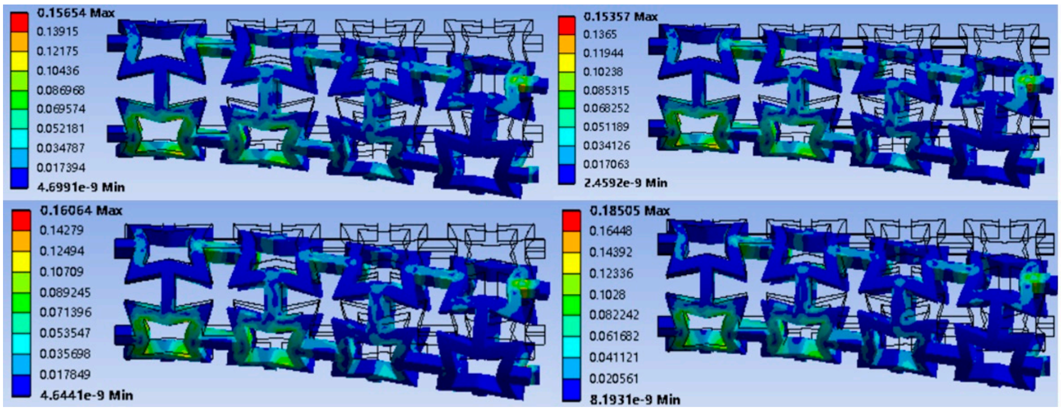

Planar Porosity 60%

(c)

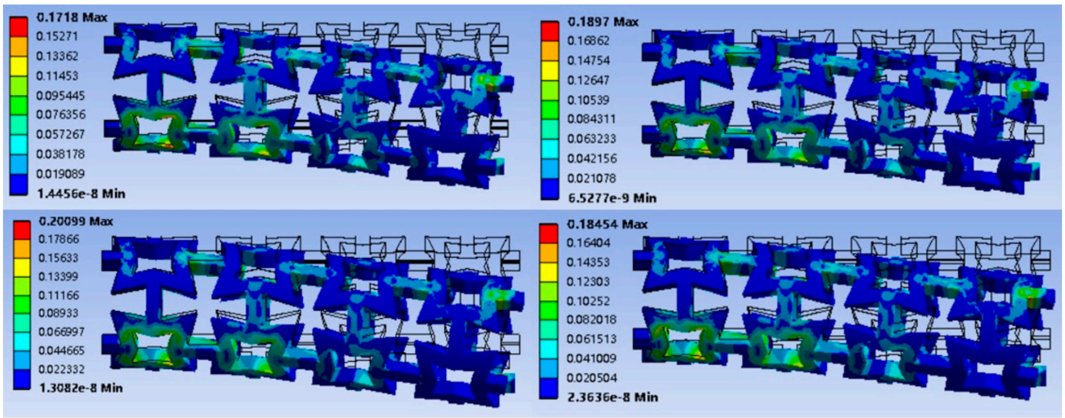

Planar Porosity 55%

(d)

Figure.S7 Single-side bending simulation equivalent stress of the Four-Angled Star-Shaped Stent under different planar porosity and wall thickness combinations. From left to right and top to bottom, wall thicknesses are 1.0 mm, 1.2 mm, 1.4 mm, and 1.6 mm

Figures.S8-12 present the simulation results of radial compression for five stent designs under different combinations of planar porosity and wall thickness. The detailed simulation procedures are described in the Materials and Methods section.

Taking **Figure.S8** as an example, **Figure.S8(a)** corresponds to the flat-edge–arc concave composite stent with a planar porosity of 70%. The four quadrants represent different wall thicknesses: 1.0 mm (upper left), 1.2 mm (upper right), 1.4 mm (lower left), and 1.6 mm (lower right).

Similarly, **Figures.S8(b), (c), and (d)** correspond to planar porosities of 65%, 60%, and 55%, respectively, with the same wall thickness distribution as in Figure.S8(a).

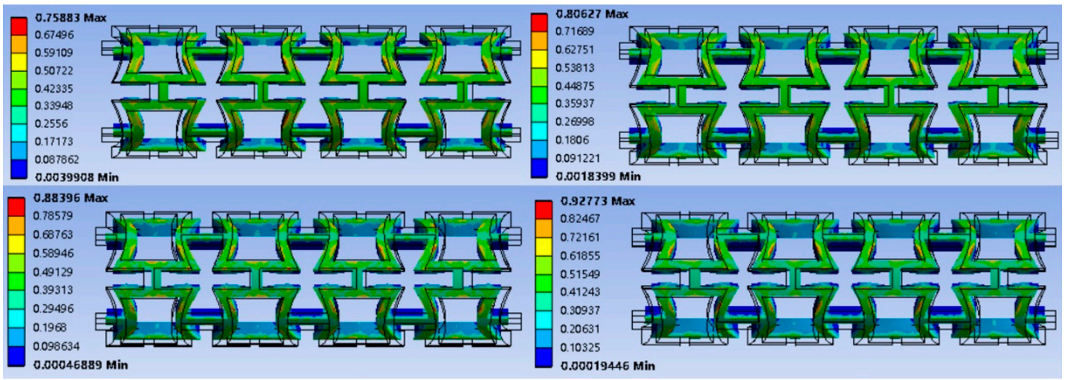

Planar Porosity 70%

(a)

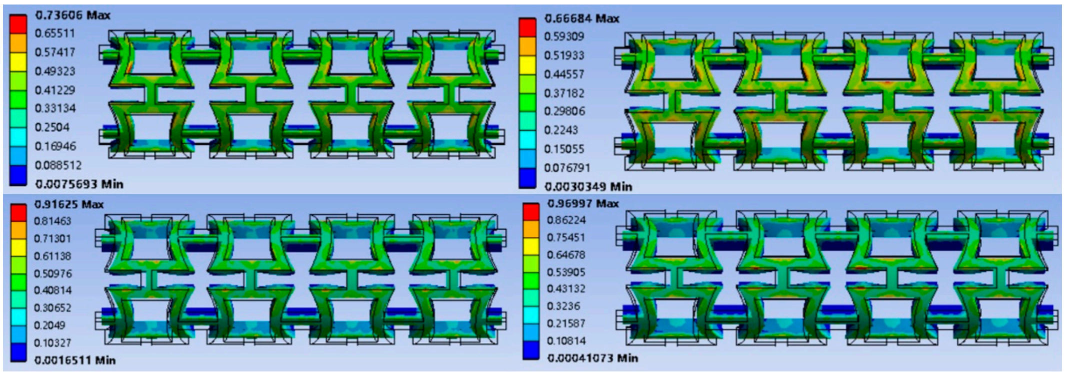

Planar Porosity 65%

(b)

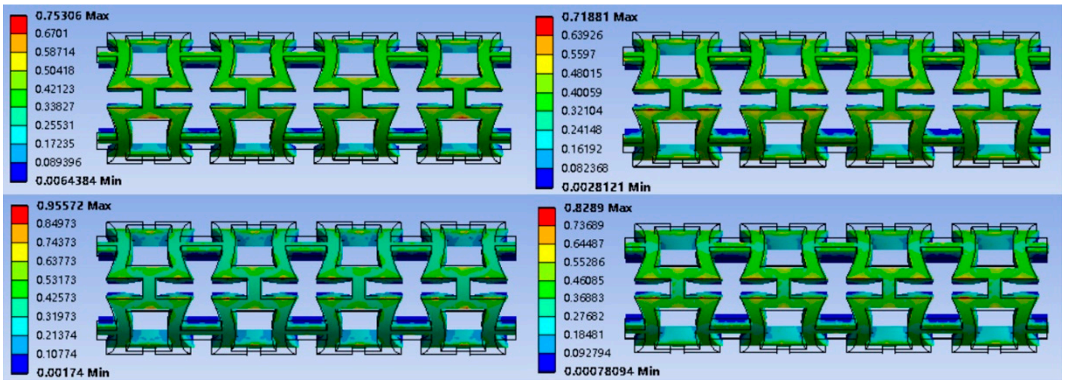

Planar Porosity 60%

(c)

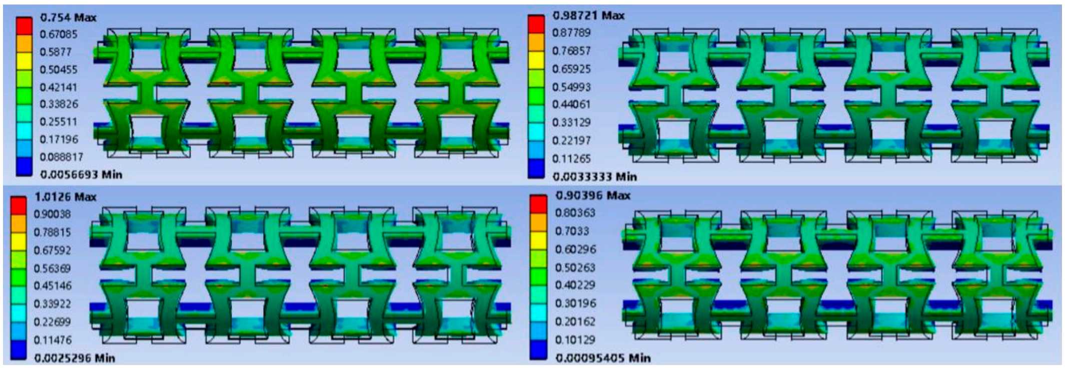

Planar Porosity 55%

(d)

Figure.S8 Equivalent stress distribution under radial compression simulation of the **Flat-Edge and Arc Composite Concave Stent** with varying planar porosity and wall thickness. From left to right and top to bottom, the wall thicknesses are 1.0 mm, 1.2 mm, 1.4 mm, and 1.6 mm

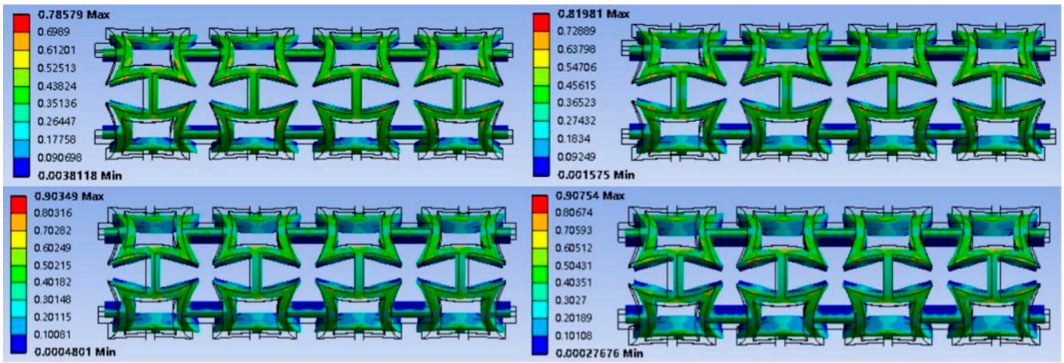

Planar Porosity 70%

(a)

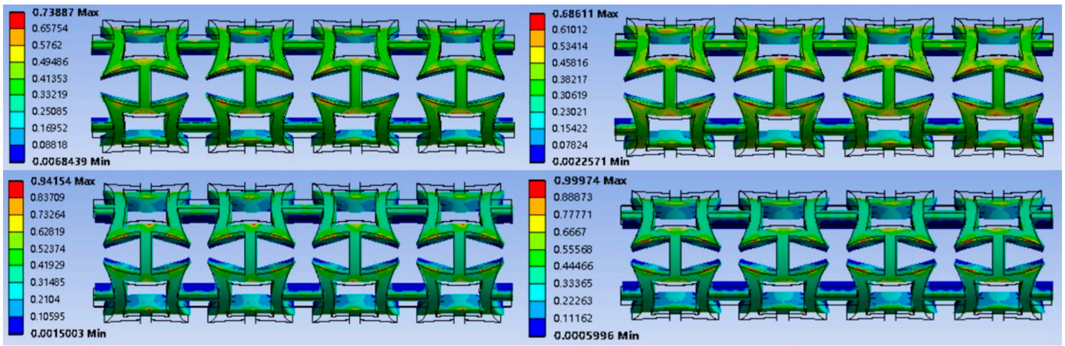

Planar Porosity 65%

(b)

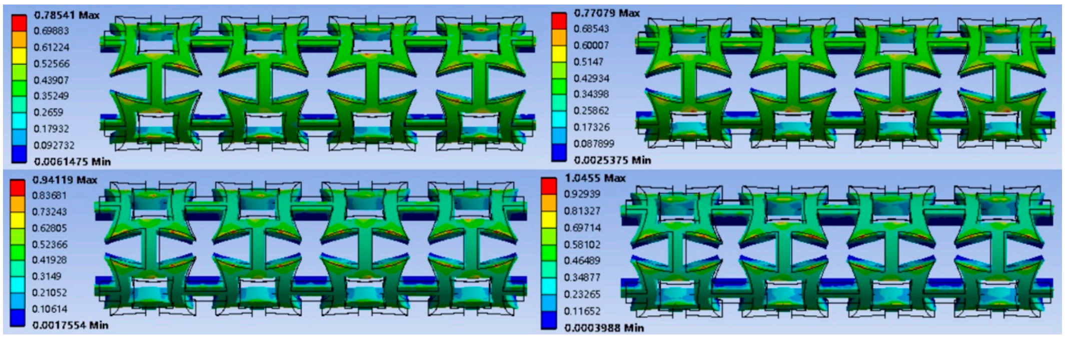

Planar Porosity 60%

(c)

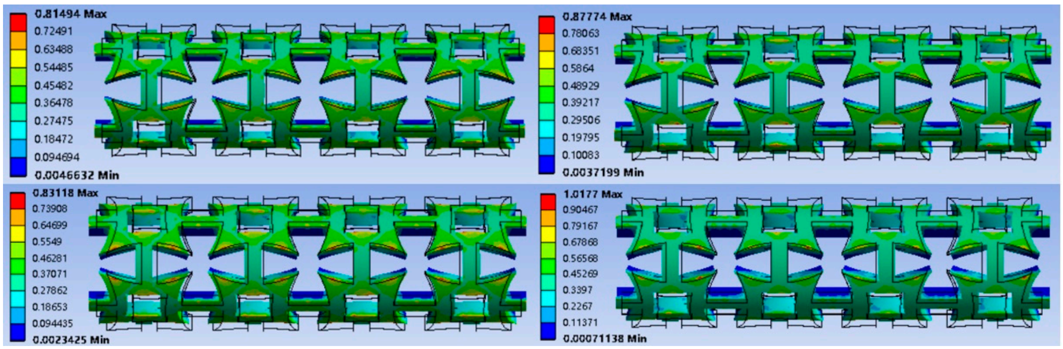

Planar Porosity 55%

(d)

Figure.S9 Equivalent stress distribution under radial compression simulation of the Fully Smooth Bidirectional Concave Stent with varying planar porosity and wall thickness. From left to right and top to bottom, the wall thicknesses are 1.0 mm, 1.2 mm, 1.4 mm, and 1.6 mm

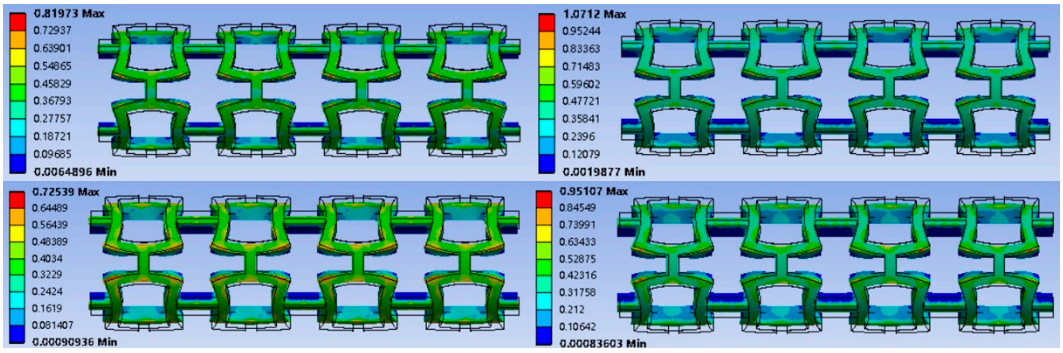

Planar Porosity 70%

(a)

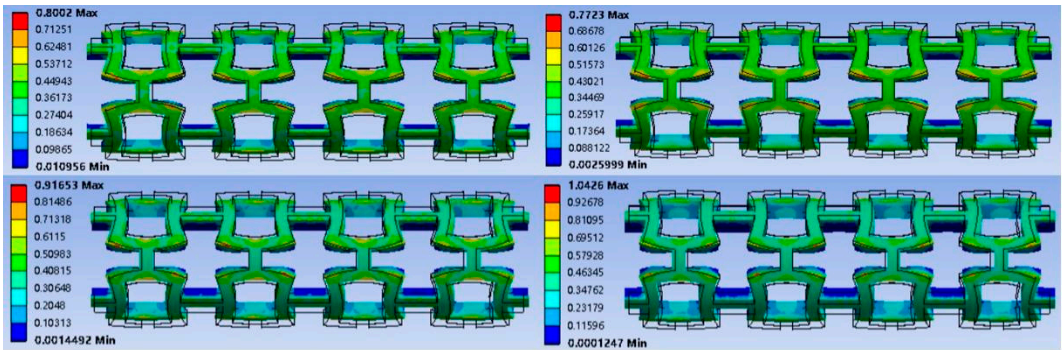

Planar Porosity 65%

(b)

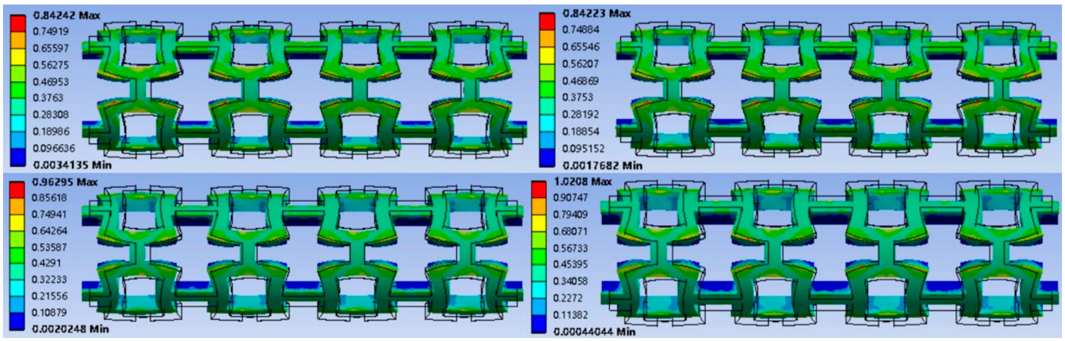

Planar Porosity 60%

(c)

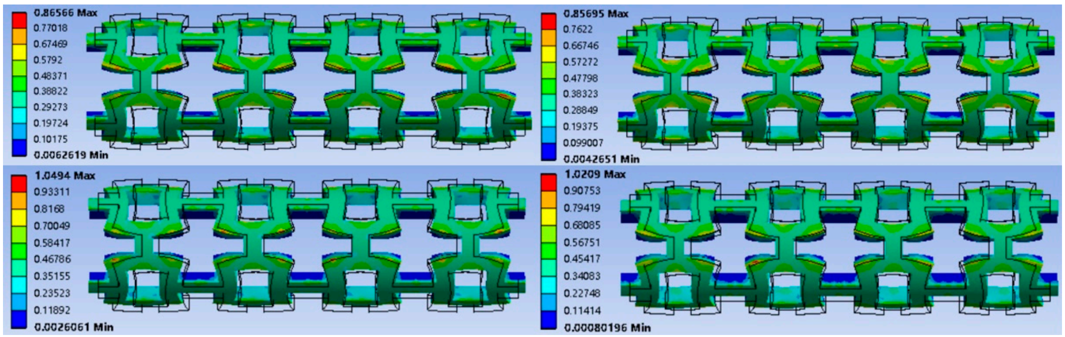

Planar Porosity 55%

(d)

Figure.S10 Equivalent stress distribution under radial compression simulation of the Concave-Convex Arc Composite Stent with varying planar porosity and wall thickness. From left to right and top to bottom, the wall thicknesses are 1.0 mm, 1.2 mm, 1.4 mm, and 1.6 mm

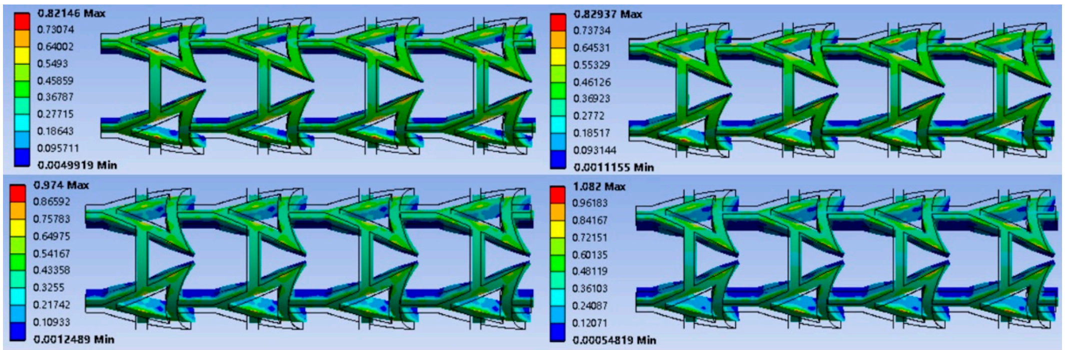

Planar Porosity 70%

(a)

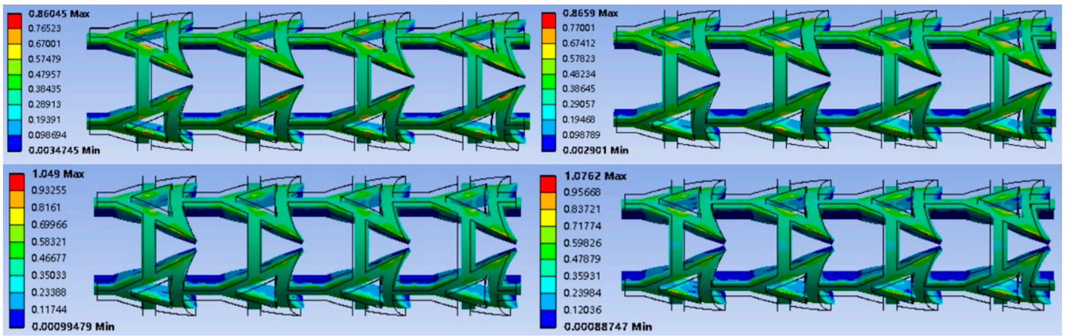

Planar Porosity 65%

(b)

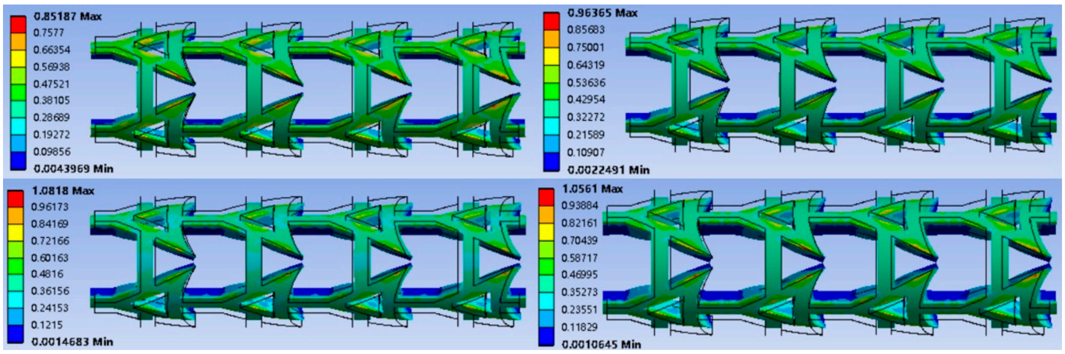

Planar Porosity 60%

(c)

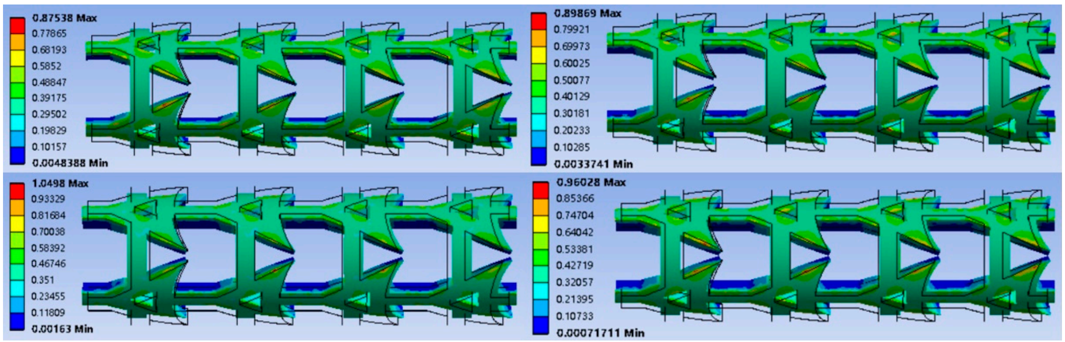

Planar Porosity 55%

(d)

Figure.S11 Equivalent stress distribution under radial compression simulation of the Unidirectional Arc Concave Stent with varying planar porosity and wall thickness. From left to right and top to bottom, the wall thicknesses are 1.0 mm, 1.2 mm, 1.4 mm, and 1.6 mm

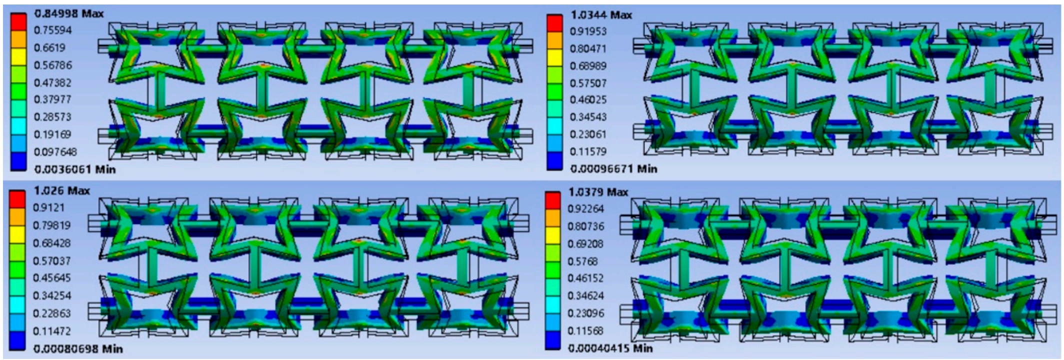

Planar Porosity 70%

(a)

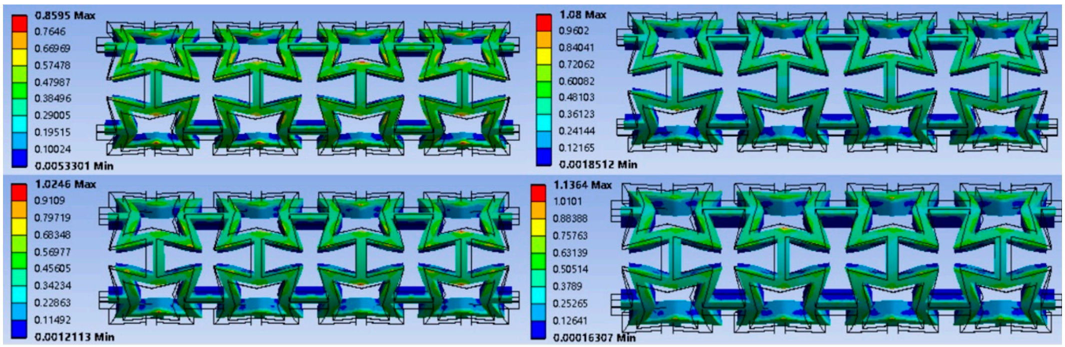

Planar Porosity 65%

(b)

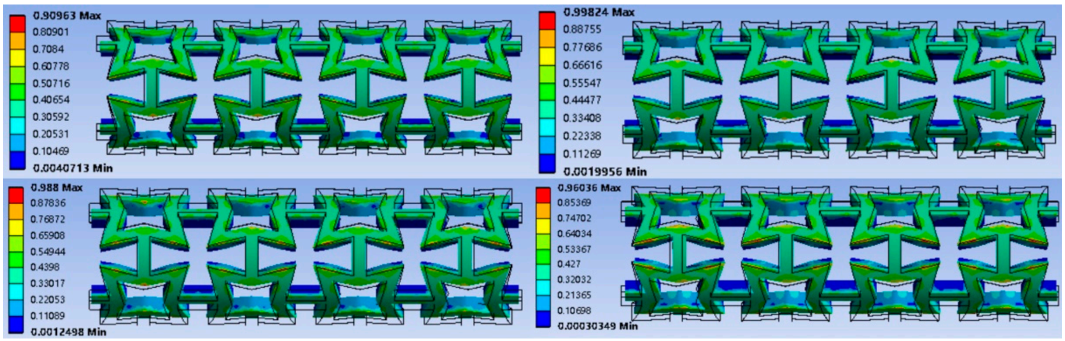

Planar Porosity 60%

(c)

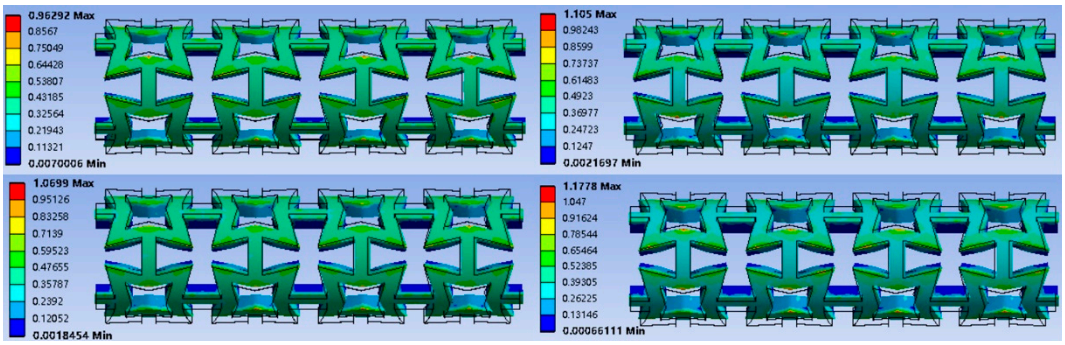

Planar Porosity 55%

(d)

Figure.S12 Equivalent stress distribution under radial compression simulation of the Four-Angled Star-Shaped Stent with varying planar porosity and wall thickness. From left to right and top to bottom, the wall thicknesses are 1.0 mm, 1.2 mm, 1.4 mm, and 1.6 mm
